# Supplementary material for: Is an artificial limb embodied as a hand? Brain decoding in prosthetic limb users
Source: PLoS Biol. 2020 Jun 8;18(6):e3000729. doi: 10.1371/journal.pbio.3000729 (PMC7302856; doi:10.1371/journal.pbio.3000729)
Supplement: S1 Text — Additional analyses conducted on ‘hand’ and ‘tool’ ROIs generated from the meta-analysis tool Neurosynth. ROI, region of interest (DOCX) [file pbio.3000729.s001.docx]

**S1 Text - Supplementary results: ‘Hand’ and ‘Tool’ ROI analysis**

To demonstrate that our results are not specific to the ROI definition used here for EBA, we’ve conducted the same analysis in ‘hand’ and ‘tool’ ROIs generated from the meta-analysis tool Neurosynth [1] (<https://neurosynth.org/analyses/terms/hand/>, <https://neurosynth.org/analyses/terms/tools/>). Using the association maps for the words: ‘hand’ and ‘tools’, ROIs were defined by using all significant voxels within the OTC (see Methods for definition of OTC). It is important to note that there is some partial overlap between the ‘hand’ and ‘tools’ ROIs, particularly in the left hemisphere (see figure S4). This is a known limitation of the spatial distribution of hand- and tool-selectivity in OTC, which is the primary reason we opted for an a priori independent ROI definition of EBA. Furthermore, as these ROIs were transformed from standard space and were not localised for each individual subject, the probabilistic ROI approach suffers from potential for reduced statistical power.

Both the ‘hand’ and ‘tool’ ROIs showed no significant visual hand-similarity (embodiment) group differences or correlation with prosthesis usage (p>0.2 for all). The ‘hand’ ROI showed a significant prosthesis-similarity (categorisation) group difference (t(54)=-2.84, p=0.01), with prosthesis users showing a stronger categorisation effect, and a trend toward significance for the correlation with prosthesis usage (r(30)=0.32, p=0.08). The ‘tools’ ROI did not show a significant categorisation prosthesis-similarity group effect (t(54)=-0.56, p=0.58), but showed a significant correlation with usage (r(30)=0.40, p=0.03).

Furthermore, when performing the ‘own prosthesis’ analysis, the Neurosynth ‘hand’ ROI showed a significant shift of the own prosthesis away from hands (t(25)=6.70, p<0.001) but not away from tools (t(25)=1.78, p=0.087). Neurosynth’s ‘tool’ ROI showed a significant shift of the own prosthesis away from both hands (t(25)=4.19, p<0.001) and tools (t(25)=4.3, p<0.001). This additional analysis confirms that our key finding in OTC are relatively robust.

**References:**
1. Yarkoni T, Poldrack RA, Nichols TE, Van Essen DC, Wager TD. Large-scale automated synthesis of human functional neuroimaging data. Nat Methods. 2011. doi:10.1038/nmeth.1635
