# Supplementary figures and images for: Is an artificial limb embodied as a hand? Brain decoding in prosthetic limb users
Source: PLoS Biol. 2020 Jun 8;18(6):e3000729. doi: 10.1371/journal.pbio.3000729 (PMC7302856; doi:10.1371/journal.pbio.3000729)

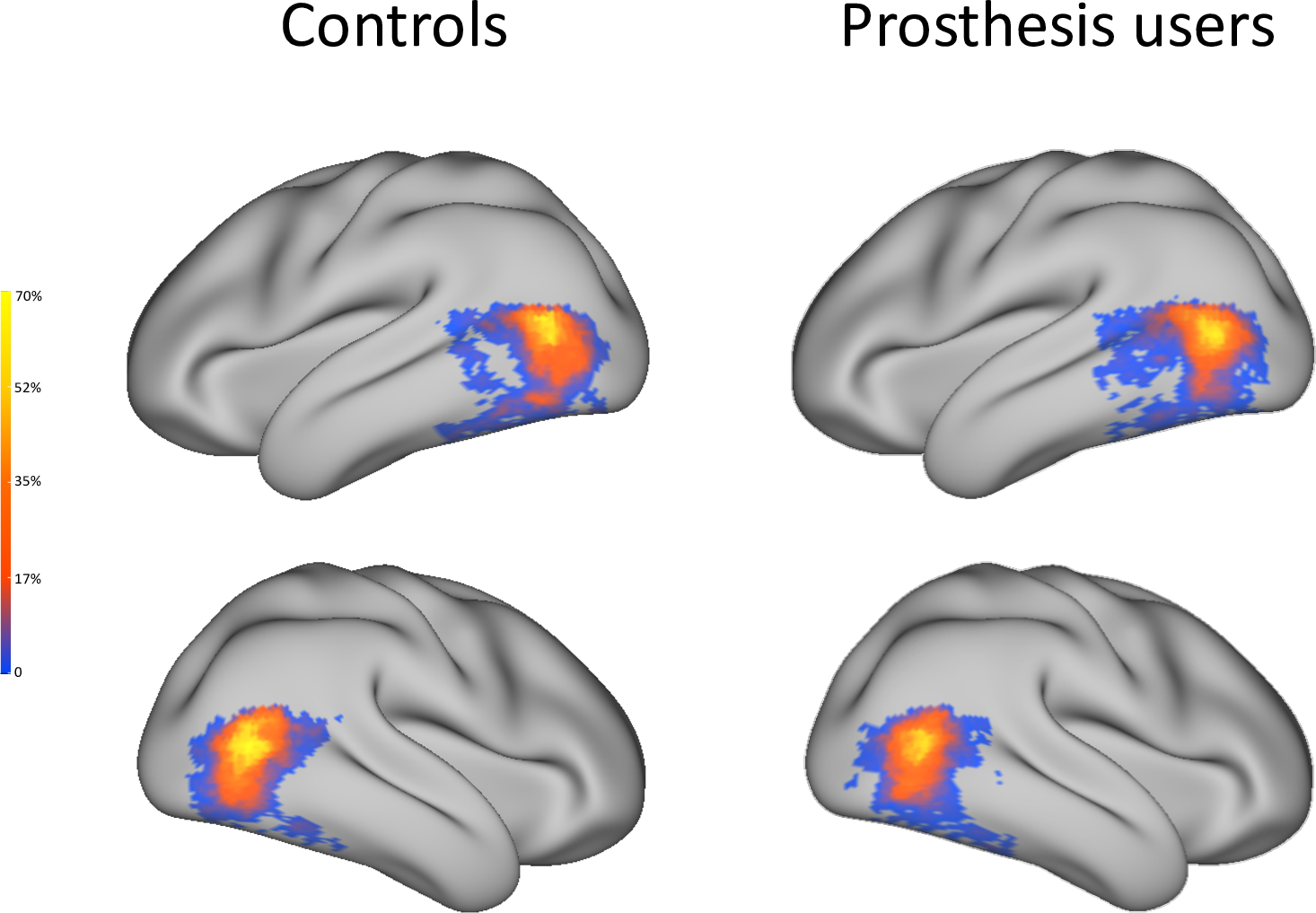

Supplement: S1 Fig — All individual visual ROIs were superimposed per group, yielding corresponding probability maps. Warmer colours represent voxels that were included in greater numbers of individual ROIs. Data used to create this figure can be found at https://osf.io/4mw2t/. ROI, region of interest (TIF) [file pbio.3000729.s006.tif]

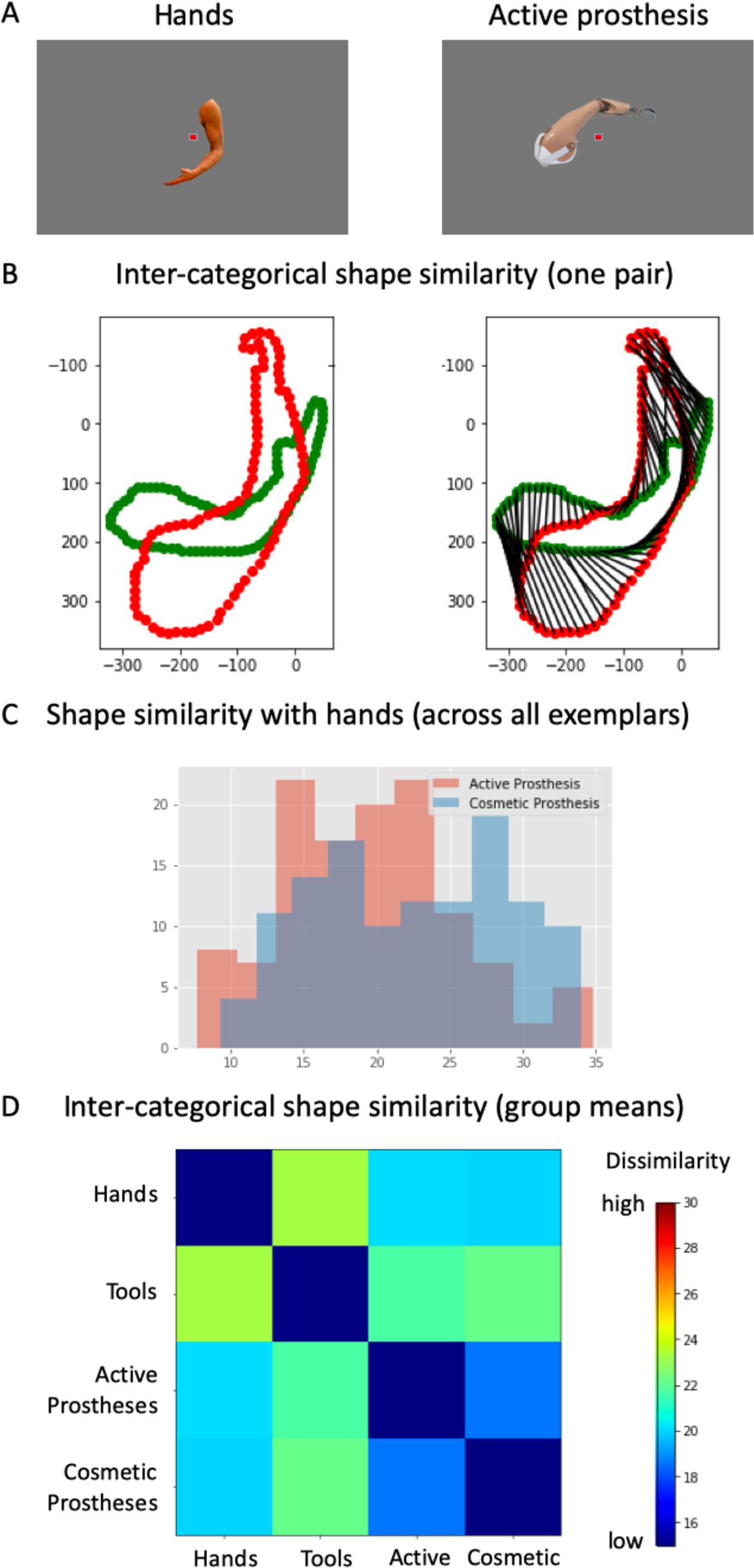

Supplement: S2 Fig — (A) Two exemplars from the ‘hand’ and ‘active prosthesis’ categories. (B) All exemplars shown to each individual participant were submitted to a visual shape similarity analysis (Belongie and colleagues, 2002), in which intercategorical pairwise shape similarity was assessed. (C) A histogram showing intercategorical similarity from one participant’s shown cosmetic (blue) and active (red) prosthesis exemplars, with respect to hand exemplars (all exemplars are available on https://osf.io/kd2yh/). As demonstrated in this example, these dissimilarity ranges were largely overlapping. (D) This intercategory dissimilarity analysis was repeated for each of the participants (based on the specific prostheses exemplars shown to them), and mean histogram values were averaged. As indicated in the resulting matrix, cosmetic and active prostheses did not show strong differences in similarities, on average. This is likely due to the wide range of exemplars/shapes used in the study data set. Data used to create this figure can be found at https://osf.io/4mw2t/. (TIF) [file pbio.3000729.s007.tif]

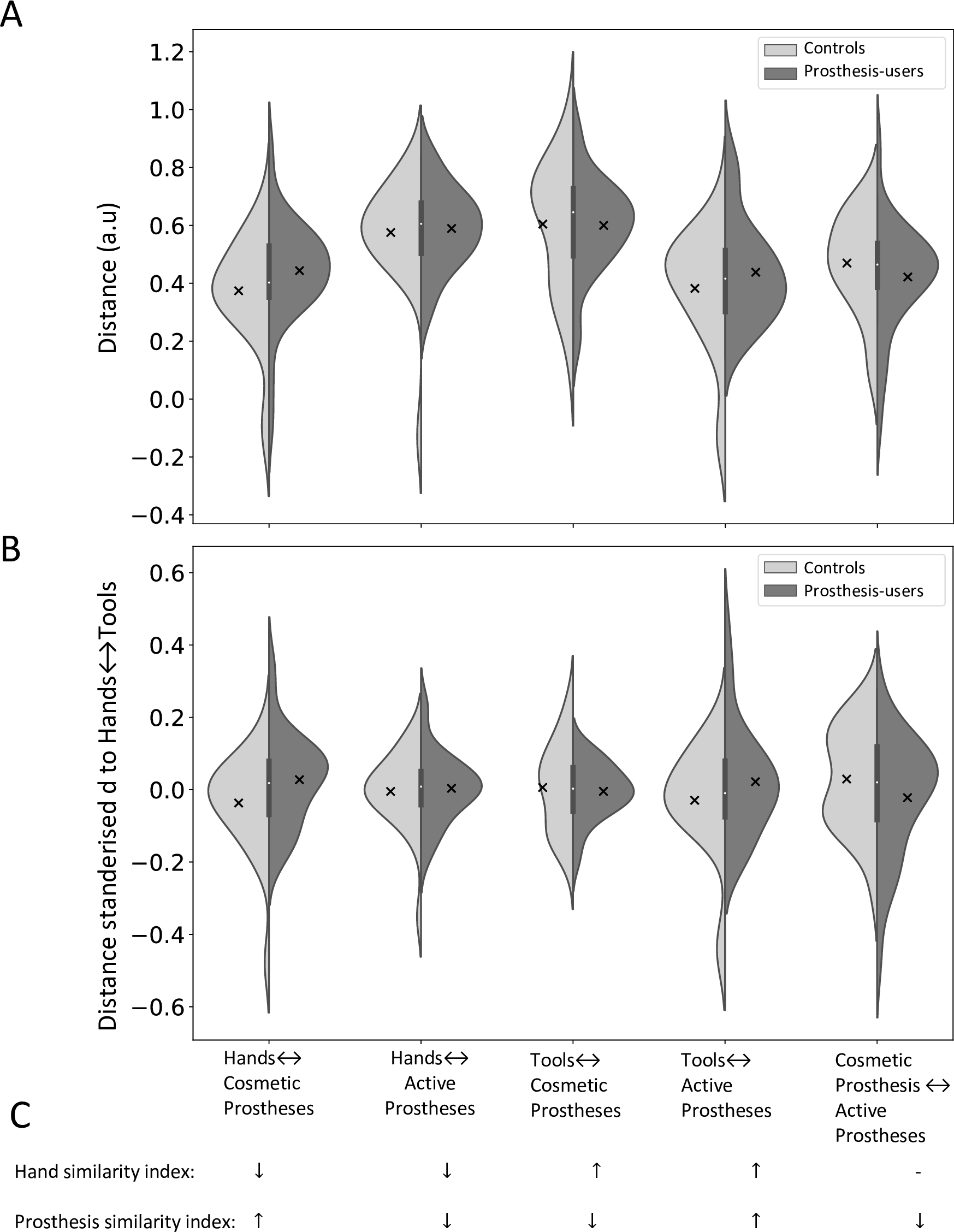

Supplement: S3 Fig — (A) Pairwise distances between patterns of activations of hands, cosmetic prostheses, active prostheses, and tools. In the labels, ‘↔’ indicates the distance between a pair of conditions. Within the plot, x indicates the group’s mean. (B) same as panel A, only with each distance standardised by the individuals’ distances between hands and tools. (C) A table illustrating the direction of the effect predicted by each index. Data used to create this figure can be found at https://osf.io/4mw2t/. EBA, extrastriate body-selective area (TIF) [file pbio.3000729.s008.tif]

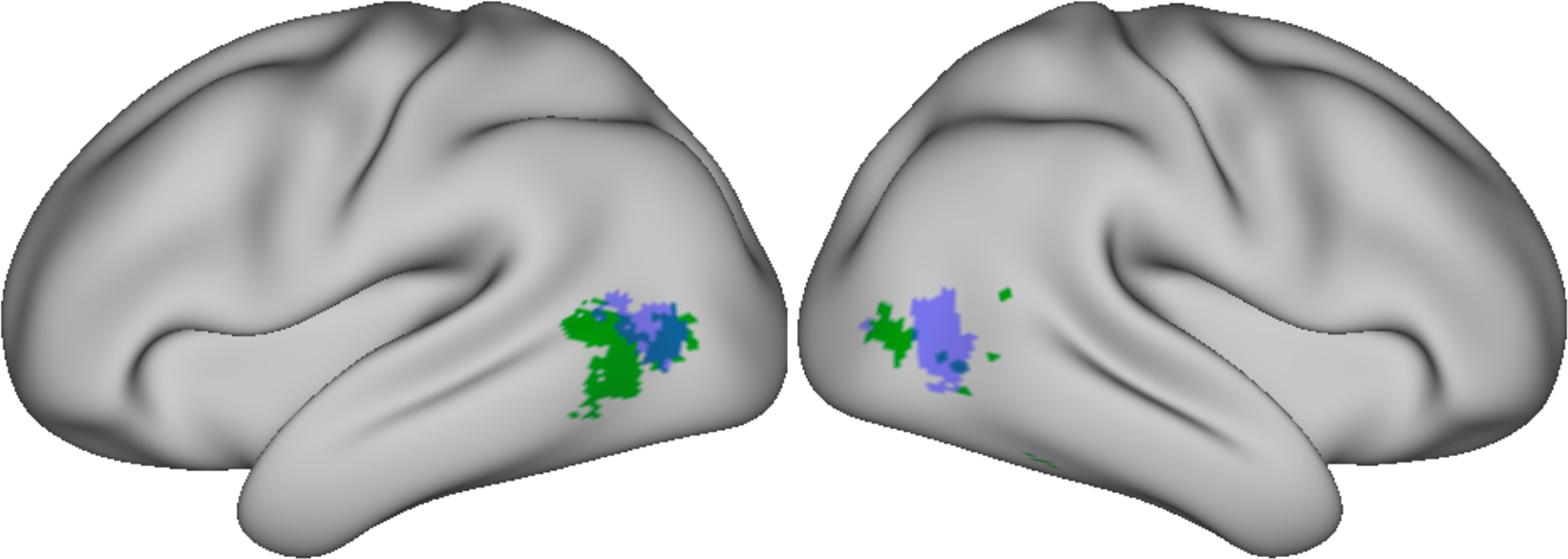

Supplement: S4 Fig — Using the association maps for the words: ‘hand’ (blue) and ‘tools’ (green), ROIs were defined by using all significant voxels within the OTC. These ROIs are projected on inflated brain for visualisation. Surface and volume masks can be found at https://osf.io/4mw2t/. OTC, occipitotemporal cortex; ROI, region of interest (TIF) [file pbio.3000729.s009.tif]

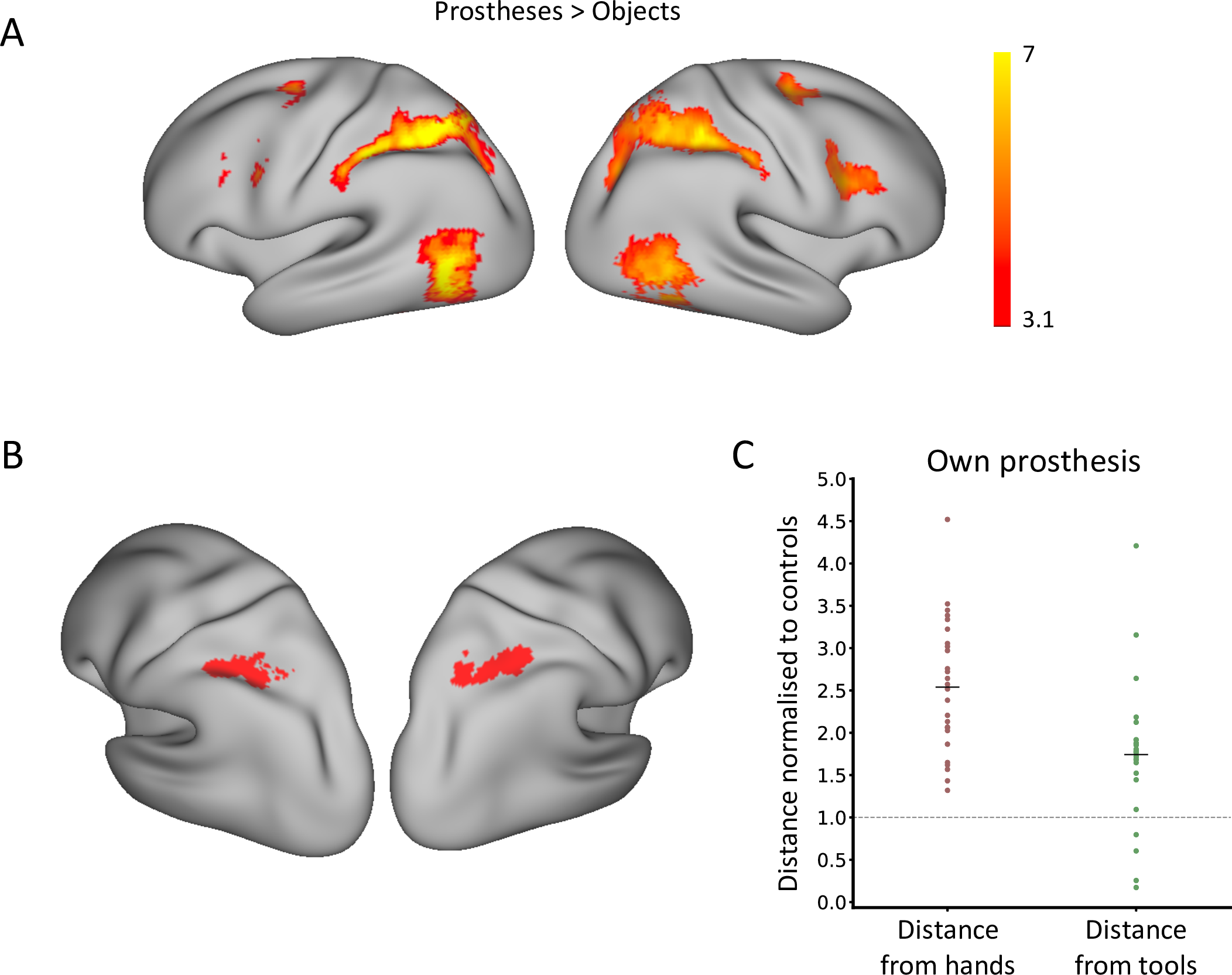

Supplement: S5 Fig — (A) Univariate activations in prosthesis users. Results of the group level univariate contrast of (Active Prosthesis + Cosmetic Prosthesis) > Objects show that at the group level the IPS is also activated. (B) The IPS region of interest was taken from the Juelich Histological Atlas (30% probability of hIP1, hIP2, and hIP3). (C) Hand (left) and tool (right) distances from users’ ‘own’ prosthesis in IPS. Individual distances were normalised by the controls’ group mean distance, depending on the visual features of the ‘own’ prosthesis (hand-likeness). A value of 1 indicates similar hand/tool distance to controls. Users showed significantly greater distances between their own prosthesis and hands (t(25) = 10.11, p < 0.001) contrary to the embodiment hypothesis. A significant increase in the distance of the ‘own’ prosthesis from tools was also observed (t(25) = 4.62, p < 0.001). Data used to create this figure can be found at https://osf.io/4mw2t/. hIP, human intraparietal; IPS, intraparietal sulcus. (TIF) [file pbio.3000729.s010.tif]
