# Supplementary material for: Is an artificial limb embodied as a hand? Brain decoding in prosthetic limb users
Source: PLoS Biol. 2020 Jun 8;18(6):e3000729. doi: 10.1371/journal.pbio.3000729 (PMC7302856; doi:10.1371/journal.pbio.3000729)
Supplement: Attachment — Submitted filename: Rebuttal_drat3.docx [file pbio.3000729.s011.docx]

Dear Professor Gasque,

Thank you for providing us with the opportunity to improve our manuscript. In our revisions, we have comprehensively addressed the issues raised by the reviewers, as elaborated in the point-by-point responses below (original comments in blue italics, our responses in black and text inserts in black italics). In particular, as you recommended, we paid special attention to the common concern about using visual areas and passive assessments. In the revised manuscript we have addressed this issue from both theoretical and practical perspectives. Practically, in addition to new analysis in motor cortex, in the revised manuscript we provide ***further results from parietal cortex, showing that the representation of one’s own prosthesis is significantly more dissimilar from that of a hand***, in comparison to controls hand-prosthesis representational similarity. This provides further strengthening to our key results and main interpretation that in terms of visual embodiment, amputees do not represent a prosthesis like a hand. Conceptually, we now elaborate why visual embodiment in OTC provides an ideal testing bed for the question, whether prostheses are represented like hands or tools. As we elaborate below, this is due to ***the crucial role of visual processing in probing embodiment, as well as the role of vision in prosthesis usage***. In addition, the specific brain area we are focusing on has been shown to ***respond to hand-related motor inputs*** and possess ***unique architecture to connect it with other key sensorimotor regions*** specifically used for hand motor control*.* Considering this, and the fact that this area is known for processing hands and tools, we have a strong case to support our research focus. Finally, in our revised manuscript we clarify that although we are testing a key model for embodiment, other sensory systems might produce different solutions to the problem – how to represent an artificial limb. Nevertheless, considering the massive prominence of the embodiment model across the multiple disciplines engaged with prosthesis design, usage and representation. we feel strongly that ***even if not offering an exhaustive answer to the question ‘how the human brain represents artificial limbs?’, our findings provide an important alternative that needs to be considered***. We hope that our Report will provide a ***new standard, in terms of methodological care and sample size***, for how to tackle this important question in future research aiming at different brain areas/systems. We feel confident that our revisions have improved the manuscript and will satisfy the reviewers.

Reviewers remarks

*Reviewer #1: The authors present a study on the fMRI activation in the occipitotemporal cortex (OTC) related to different prostheses types. They found that prosthesis-use let to OTC fMRI activations more similar to those of tools than to hands, and that greater daily-life prosthesis usage correlated with greater prosthesis (tool) categorisation, documenting use-dependent plasticity within the OTC. Overall, the manuscript is well written, the methods clear and the results interesting, but there a few issues that need to be addressed.*

*1.1 It is stated that “Two hypotheses were tested: the embodiment hypothesis, assessing whether prosthetic limbs are in fact represented as hands (and not tools); and, the categorization hypothesis, assessing whether a new ‘prosthesis’ category has formed.” It is unclear what predictions these hypotheses make about the presented outcome measures (representational similarity as measured as OTC fMRI activation). Please clarify in the introduction.*

We thank the reviewer for bringing this to our attention. We now highlight these predictions, on which we elaborate at the beginning of the results section, as part of the introduction:

Introduction (4^th^ paragraph):

*“To provide distinct predictions for each of these hypotheses, we studied representational similarities between hand, tools, and upper limb prosthetics images (both cosmetic - designed to resemble hand appearances; and active – designed to afford a grip, e.g. a “hook”; Figure 1A) and compared prosthesis users to controls. Broadly speaking, the visual hand embodiment hypothesis predicts that compared to controls, the various prosthesis conditions in prosthesis-users will be more similar to hands than to tools (notice that this prediction also allows us to test the inverse prediction – that prostheses are represented more like tools in one-handers). The categorisation hypothesis predicts that in prosthesis-users, the prosthesis conditions will be more similar to each other relative to hands and tools.”*

1.2 "In other words, prosthesis users do not, on average, ‘embody’ prostheses as hands more than controls." The lack of a statistically significant difference in the hand-similarity index in prosthesis users relative to controls is not a good argument in favour of the equality of this measure, i.e., an argument against the embodiment hypothesis. Please provide equivalence test to support this statement, or at least a power analysis.

We fully take this point. We have now added a Bayesian analysis to further confirm this non-significant result reflects a null finding. Later on, in the results section, we also directly compared the correlation between the embodiment index and the categorisation index, to demonstrate that categorisation provides a better model of our data. Nevertheless, we have removed this sentence from the revised manuscript, as we appreciate it does not fit this paragraph.

Results (2^nd^ section, 1^st^ paragraph):

*“When comparing hand-similarity indices based on the multidimensional distances across participant groups, we found no significant differences (t(54) = 0.47, p=0.64; Figure 3A). A Bayesian t-test provided substantial evidence in support of the null hypothesis (BF=0.2), i.e. that on average amputees do not represent an unfamiliar prosthesis more similarly to a hand than controls.”*

1.3 What was the rationale for choosing to examine BOLD activity in the extra striate region of the OTC? What about motor cortex representations? Were any other regions examined? What were the findings?

We thank the reviewer for providing us an opportunity to elaborate on this key point. We have now revised the introduction and discussion to better reflect the a priori conceptual advantages of studying visual embodiment in EBA.

First and foremost, embodiment is a multisensory experience, which is predominantly measured by means of visual manipulations. For example, in the rubber hand illusion, vision is used to present both the rubber hand and the brush that is ‘capturing’ the felt touch. Of particular importance here, visual embodiment is conceived by some as a gateway for the sense of bodily ownership (Tsakiris, 2010). Moreover, prosthesis usage is highly visually-guided (Antfolk et al., 2013). As such, research of prosthesis embodiment should build on the importance of visual experience in daily prosthesis usage. An additional, more minor, consideration for us was that sensorimotor embodiment would be difficult to test since in congenital one-handers, the sensorimotor homunculus is architecturally changed compared with controls (Hahamy et al., 2017; Hahamy & Makin, 2019; Wesselink et al., 2019). Therefore, differences observed in these areas would be difficult to interpret.

Introduction (2^nd^ paragraph):

*“Embodiment is an umbrella term for the multisensory association of external objects with body representation, which engages multiple levels from both conceptual and perceptual perspectives (see Discussion). From a neural standpoint, embodiment is defined by the successful allocation of brain resources, originally devoted to controlling one’s own body, to represent and operate external objects [17]. Visual embodiment is particularly relevant for studying prosthesis representation. This is because prosthesis usage is highly visually guided [18], and because visual embodiment has been suggested as a gateway for the sense of bodily ownership [19], a desired feature of prosthesis usage [9,10]. Previous efforts to test visual prosthesis embodiment have been centred on an illusion of body ownership over the prosthesis (most commonly, the rubber hand illusion, which relies on visual manipulations), with mixed success [11,20–23].”*

EBA is a body- and hand-selective region, and is therefore suitable to testing embodiment (i.e. hand-like representation of artificial limbs). We (Orlov, Makin, & Zohary, 2010) and others (Bracci, Caramazza, & Peelen, 2015) have shown that this area selectively responds to body parts, including hands. EBA is not strictly visual, as it also contains sensorimotor attributes. We and others have shown that the region responds to hand movements (Astafiev, Stanley, Shulman, & Corbetta, 2004; Orlov, Porat, Makin, & Zohary, 2014) and passive touch (Tal, Geva, & Amedi, 2016). This is also very clearly demonstrated in blinds (Wesselink, Kikkert, Bridge, & Makin, 2018). EBA’s connectome further associates it with hand actions: it is uniquely connected to the sensorimotor hand area (Tal et al., 2016). We have recently shown that inter-modal connectivity between visual and motor hand-selective regions is modulated by the degree to which individuals use their prosthetic limb (Van Den Heiligenberg et al., 2018). This evidence indicates that this region is sensitive to daily prosthesis usage. Finally, and of particular relevance to our key research question, there is a known overlap between the hand-selective and tool-selective regions within the extrastriate area, making it an ideal brain area to test for the hypothesis that a prosthesis is represented like a tool (Bracci, Cavina-Pratesi, Ietswaart, Caramazza, & Peelen, 2012).

Introduction (3^rd^ paragraph):

*“The OTC is known to contain distinct visual representations of different object categories [30], for example: hands [31,32], tools [33], faces [34], and bodies [35]. It was previously shown to contain overlapping visual and motor body-part selective representations [31,36], and even responds to touch [37,38]. OTC’s connectome associates it with hand actions. For example, hand and body-selective visual areas uniquely connect to the primary sensorimotor hand area [37]. These characteristics qualify the OTC as an ideal candidate for studying action-related visual body-representation. We previously found that individuals who used their prosthesis more in daily lives also showed greater activity in OTC’s hand-selective visual areas when viewing images of prostheses, and greater functional coupling with sensorimotor hand areas [29]. This result demonstrates that prosthesis users are able to engage visuomotor hand-selective areas while (passively) representing a prosthesis.“*

Introduction (3^rd^ paragraph):

*“Alternatively, since the visual hand-selective regions partially overlap with hand-held tool representation [39], the observed activity gains may reflect the prosthesis being represented as a tool.“*

For these reasons, we believe this region provides a key model for addressing our question, and have thus designed the experiment to activate this region (i.e. static images). However, in the revised manuscript we also explored a few other brain regions. First, we include an additional analysis with similar finding, focused on an independent hand-selective ROI in OTC. This additional exploratory analysis demonstrates that our results are not specific to the chosen ROI (See supplementary results). Second, since the group level analysis showed significant activity in the intra parietal sulcus (IPS), in the revised manuscript we include additional exploratory analysis performed in IPS, as detailed below.

Results (5th section):

*“****Prosthesis representation beyond EBA***

*To demonstrate that our results are not specific to our ROI definition of EBA, we’ve repeated the same analysis, and found similar results, in ‘hand’ and ‘tool’ ROIs within OTC, generated from the meta-analysis tool Neurosynth [47] (see Supplementary results and Figure S4).*

*The stimuli used in the current study were specifically designed to interrogate the information content underlying prosthesis representation in body-selective regions in the OTC. Nevertheless, as demonstrated in Figure S5A, the visual stimuli also significantly activated the intraparietal sulcus (IPS), providing us with an opportunity to explore visual prosthesis representation in a dissociated brain area (though notably, this was observed less consistently within individual subjects). Since 11 of the participants did not have enough significantly activated voxels within IPS to meet our ROI criteria, we constructed an anatomical ROI based on the Juliech Histological Atlas in FSL, including human intraparietal (hIP)1-3 bilaterally [48,49] (Fig. S5B). With respect to RSA, we did not find significant differences between prosthesis users and controls when comparing both the hand- and prosthesis-similarity indices, even when wavering corrections for multiple comparisons which are customary for exploratory analysis (hand-similarity: t(54)=0.71, p=0.48; prosthesis-similarity: t(54)=-0.45, p=0.66). However, we did find that users showed significantly greater dissimilarity (greater distances), relative to controls, when comparing the representation of their own prosthesis to that of both hands and tools (hand: t(25)=10.11, p<0.001; tool: t(25)=4.62, p<0.001, corrected for 2 comparisons, see Fig. S5C). This analysis indicates that similar to the OTC, in parietal cortex too, users’ own prostheses are less similar to hands and tools, providing support in favour of the categorisation interpretation, and against the embodiment hypothesis. “*

Supplementary figure:

*“****Supplementary Figure S5. Intraparietal Sulcus (IPS) analysis.*** *(A) Univariate activations in prosthesis users. Results of the group level univariate contrast of (Active Prosthesis + Cosmetic Prosthesis) > Objects show that at the group level the IPS is also activated. (B) The IPS region of interest was taken from the Juelich Histological Atlas (30% probability of hIP1, hIP2, and hIP3). (C) Hand (left) and tool (right) distances from users’ ‘own’ prosthesis in IPS. Individual distances were normalised by the controls’ group mean distance, depending on the visual features of the ‘own’ prosthesis (hand-likeness). A value of one indicates similar hand/tool distance to controls. Users showed significantly greater distances between their own prosthesis and hands (t(25)=10.11, p<0.001contrary to the embodiment hypothesis. A significant increase in the distance of the ‘own’ prosthesis from tools was also observed (t(25)=4.62, p<0.001).”*

Lastly, we have also attempted an analysis in primary sensorimotor cortex. The rationale behind this analysis was the tight relationship previously observed between the sensorimotor and visuomotor hand regions (Tal et al., 2016; Van Den Heiligenberg et al., 2018). Univariate BOLD activity was negative and significantly different from zero for all 5 conditions, this encouraged us to run a multivariate analysis (since significant multivariate distances can be produced from negative activity (Diedrichsen, Wiestler, & Krakauer, 2013). However, as demonstrated in the table below, pairwise distances were not significantly different from zero, indicating that no reliable information could be extracted from the sensorimotor cortex using the present paradigm.

| **Univariate activity (z-statistic) in sensorimotor cortex for each condition** | | | | | |
| --- | --- | --- | --- | --- | --- |
|  | Hands | Tools | Active Prosthesis | Cosmetic Prosthesis | Own Prosthesis |
| Controls | -1.09 | -1.14 | -1.2 | -1.02 | - |
| T-test mean $\neq$ 0 | p<0.001 | p<0.001 | p<0.001 | p<0.001 | - |
| Prosthesis Users | -1.06 | -1.37 | -1.2 | -1.2 | -1.3 |
| T-test mean $\neq$ 0 | p<0.001 | p<0.001 | p<0.001 | p<0.001 | p<0.001 |

*values are average of the z-statistic across the ROI

| **Multivariate distances (RSA) in sensorimotor cortex** | | | | | |
| --- | --- | --- | --- | --- | --- |
|  | Hands ↔ Cosmetic Prosthesis | Hands ↔ Active Prosthesis | Tools ↔ Cosmetic Prosthesis | Tools ↔ Active Prosthesis | Cosmetic Prosthesis ↔ Active Prosthesis |
| Controls | -0.03 | 0.03 | 0.05 | 0.02 | 0.03 |
| T-test mean $\neq$ 0 | p=0.4 | p=0.32 | p=0.25 | p=0.57 | p=0.48 |
| Prosthesis Users | 0.04 | 0.04 | -0.02 | 0.01 | 0.07 |
| T-test mean $\neq$ 0 | p=0.26 | p=0.17 | p=0.63 | p=0.87 | p=0.04 |

*values are cross-nobis (LDC) distances; ↔ indicates pairwise distances.

1.4 The lack of a linear relationship between prosthesis use and the hand similarity index is contrasted against the strong linear relationship between prosthesis use and the prosthesis similarity index. The latter relationship doesn't need to be driven by the separation of prosthesis from hand representations, because the former relationship would have reflected this too. This implies that the strong linear relationship between ‘prosthesis use’ and ‘prosthesis similarity index’ is driven by the distance between prosthesis representations. Please disentangle the distance between prosthesis representations from the distance between other prosthesis and hand representations.

The prosthesis similarity index involves multiple pairwise distances, as illustrated in figure 4A, with outward facing errors indicating divergence and inward facing arrows indicating convergence.

In addition to the divergence of the cosmetic prosthesis away from the hand and towards the tool categories, the prosthesis similarity index also involves a divergence of the functional prosthesis in the opposite direction, as well as a component of each of the prostheses converging towards each other. As such, it should not be viewed as an opposite, but rather an alternative model to the embodiment model. Importantly, in our analysis we never compare between the two indices values directly. Instead, we use them as models to capture everyday prosthesis usage, and only directly compare the resulting model fits (r square). To address the reviewer’s query, we have now performed a direct correlation between prosthesis usage and the distance between the prostheses, which was not significant (r(30)=-0.063, p=0.73). We have also recalculated the hand similarity index with the relationship between the two prostheses added as a negative contributor [using the following formula: 3*(Tools↔CosmP + Tools↔ActP) – 2* (Hands↔CosmP + Hands↔ActP +ActP↔CosmP). Similar to our original results, we found no significant group effect t(54)=-0.41, p=0.68 and no significant correlations with prosthesis usage: r(30=0.09, p=0.61). In the revised manuscript we clarified on our approach, in addition to a quantification and visualisation of all 5 distances used to calculate the indices for better transparency of the results.

Results (2^nd^ section, 5^th^ paragraph):

*“The two indices for hand- and prosthesis-similarity are not statistically independent, and as such we were not able to compare them directly. However, we could use them as a model for predicting daily life prosthesis usage. Comparing the correlation between prosthesis usage and the indices for the two hypotheses (embodiment and categorical) revealed a significant difference (z=-2.52, p=0.01). Therefore, at least when considering unfamiliar prostheses, the prosthesis-similarity index is a better predictor of prosthesis usage than the hand-similarity index.”*

Supplementary figure:

***“Supplementary Figure S3. Pairwise distances in EBA.*** *(A) Pairwise distances between patterns of activations of hands, cosmetic prostheses, active prostheses, and tools. In the labels, ↔ indicates the distance between a pair of conditions. Within the plot, x indicates the group’s mean. (B) same as (A) only with each distance standardised by the individuals’ distances between hands and tools. (C) A table illustrating the direction of the effect predicted by each index****.****”*

1.5 "Therefore, our results show that neural embodiment does not predict successful adoption of wearable technology by the human brain." The authors found that prosthesis use correlates negatively with the prosthesis similarity index (“the degree to which the prostheses representation moved away from their natural categories (hands for cosmetic prostheses and tools for active prostheses) and towards one-another”). Doesn't this imply a negative correlation between neural embodiment (representational separation from hands) and adoption of the technology (prosthesis use)?

We thank the reviewer for making this very important point, since upon re-reading the manuscript we realise that we’ve been implying that the two models provide opposite evidence, where in fact (as mentioned above) this is not the case. Therefore, the correlation between the prosthesis similarity index and prosthesis use cannot be interpreted as an anti-embodiment effect. To support our statement that embodiment does not predict successful prosthesis usage we provide converging evidence from the hand-similarity index with respect to both unfamiliar prostheses, and individual’s own prosthesis (as elaborated above, we now provide further evidence from parietal cortex to further consolidate this latter result). Therefore, we suggest that prosthesis categorisation provides a better model for describing group and inter-individual differences in prosthesis users. We have now made changes in the discussion to better explain the relationship between the two models. Together with the revisions we suggested above (1.4), we hope that this issue is now better clarified.

Discussion (1^st^ paragraph)

*“We pitted the embodiment theory against another prominent theory - the categorisation theory – which is well validated in neuroscience [40] (as detailed below), but to our knowledge hasn’t been explored for wearable devices. While not directly opposing, both theories generate different predictions on how a prosthesis should be represented in the brain of its user. Contrary to the embodiment theory, we found that prosthesis users represented their own prosthesis unlike a hand. For unfamiliar prostheses, users formed a prosthesis category, distinct from their natural (existing) categories (hands and tools), as demonstrated in controls. Importantly, this shift scales with usage, such that people who use their prosthesis more in daily life show more independence of the prosthesis category within the hand-tool axis. When comparing the two models’ success in explaining inter-individual differences between prosthesis users, we find that the prosthesis category model was significantly more correlated with prosthesis usage. This indicates that for visual representation of unfamiliar prostheses, categorisation provides a better conceptual framework than embodiment. Together with preliminary results showing that prosthesis users exhibited a less hand-like representation of their own prosthesis in parietal cortex, our results collectively show that neural visual embodiment does not predict successful adoption of wearable technology by the human brain. Despite benefiting from hand-selective cortical resources [29], the prosthesis is not ‘embodied’ into people’s visual hand representation. However, since the experimental design and analysis we implemented were a priori focused on visual hand embodiment in OTC, it is possible that other brain areas might find a different representational ‘solution’ to support the daily use of an artificial limb.”*

1.6 "Successful prosthesis usage was predicted not by embodiment (hand-similarity),
but by a more distinct categorical representation of artificial limbs." What do you mean by successful prosthesis use? How did you measure prosthesis success rate? Unclear, please reword or explain.

We apologies for not clarifying this important point in advance. As elaborated in our methods section, prosthesis usage was assessed by combining two, highly correlated, measurements of daily usage: prosthesis wear frequency and a Prosthesis Activity Log, both measured using questionnaires. Successful prosthesis usage means a greater score of this combined measurement. As such, successful prosthesis usage could mean either more frequent wear-time and/or better utility in daily tasks. Given these measures are highly correlated (in the present study: r(30)=.84; p<.001), successful prosthesis usage is often characterised with both. This sentence has now been revised as follows:

Discussion (8^th^ paragraph):

*“Successful prosthesis usage, in terms of both wear-time and habit in daily life, was predicted not by visual embodiment (hand-similarity), but by a more distinct categorical representation of artificial limbs.”*

Reviewer #2: In this study Mor and Makin use multivariate pattern analysis of BOLD signals in the occipitotemporal cortex (OTC) to compare the neural representations of visually presented hands and prostheses in prosthesis-users and controls. The main result is that protheses are more clearly represented as its own category in prosthesis-user compared to controls. In other words, the OTC can better distinguish between hands and prostheses in the group that uses protheses. This is an interesting observation that is well worth reporting. The paper is well written, the statistical analyses has been conducted appropriately, and the main result is very clear. The study advances our understanding of OTC, use-dependent plasticity and the neural representation of artificial limbs in higher order visual cortex. However, I think the authors overstate importance of the present finding with respect to theories of embodiment and the development of advanced prostheses.

We have taken this criticism on board and have extensively revised the manuscript explaining more clearly the relevance of our findings for the lack of visual embodiment on embodiment research more broadly. We have also clarified our ideas regarding the implications of our research to the future design of prosthetic limbs:

Discussion (8^th^ & 9^th^ paragraph):

*“Finally, our results suggest that the relationship between prosthesis representation and usage is independent of key design and usage features of the artificial device (such as visual mimicry of the human hand) and cause of limb loss (congenital or acquired). This should inspire future efforts in neurologically-informed substitution and augmentative artificial limb design to not be strictly confined to biomimetics, a design principle that is currently highlighted in the development of substitution technology [80] (for example, the vine prosthesis [81]).*

*… Understanding whether the brain can treat a prosthesis as a hand, and whether this hand-like representation provides a real advantage for prosthesis users, will have important implications on future design and assessment of wearable technology. Considering the limitations related to our focus on visual prosthesis representation in passive settings we are currently unable to offer a sweeping answer to how the entire brain represents artificial limbs. However, our findings provide an important alternative to the highly prominent embodiment theory that needs to be considered. As such, much more research is necessary to provide a comprehensive understanding of the neural basis of successful prosthesis usage in the human brain.”*

Other weaknesses are that not all conclusions in the discussion are supported by the data and the manuscript includes some unnecessary speculations. Further, the finding that a new 'prothesis' category is formed in the OTC is not entirely novel as we know from earlier studies that expertise can lead to changes in the neural representations of categories in OTC. In sum, this is an interesting high-quality study but, in my opinion, the conceptual advance with respect to our understanding of artificial limb embodiment is somewhat limited. This article might therefore be better suited in a more specialized journal.

We are sorry if our interpretation and therefore the novelty of our results weren’t outlined clearly. We were surprised by the reviewer’s comment about our conclusions not being supported by the data. We wonder if this comment was sparked by the lumping together of our (relatively carefully worded) conclusion sentence [*To conclude, in this study we provide a novel neural correlate for the adoption of wearable technology. Successful prosthesis usage, in terms of both use-time and habit in daily life, was predicted not by visual embodiment (hand-similarity), but by a more distinct categorical representation of artificial limbs*] with a more speculative alternative which was not meant to be regarded as a conclusion, or even an interpretation of the findings? As elaborate below (comment 2.3), to avoid any further confusion we have reframed the discussion section in the revised manuscript, and in particular our suggestion for alternative interpretations of our findings, and the limitations of our conclusions.

Finally, we wish to address the two concerns the reviewer raises with regards to the novelty of our findings, with respect to research of visual expertise and artificial limb embodiment.

Regarding the latter, to our knowledge this is one of the only studies that (A) probes prosthesis neural embodiment directly and not through subjective questionnaires or other indirect methods (B) provides the field a practical tool to test the elusive an often-ill-defined concept of embodiment.

With regards to research on visual expertise, we wish to emphasise that the novelty of our results is not exclusive to the formation of the prosthesis category in prosthesis users, which we agree nicely converges with previous findings. As we attempted to explain in our original manuscript, we think our finding provides also additional novel information that would be of interest to researchers interested in category formation in experts. First, we are studying ecological behaviour with inherent clinical relevance, which is not taught to our subjects as a visual discrimination task. Showing that the OTC functional organisation reflects daily ecological behaviour has rarely been shown before. Second, most expert studies either focus on univariate analysis which is difficult to interpret (see (Dempsey-Jones, Wesselink, Friedman, & Makin, 2019) for discussion) or only show changes of feature encoding within the category. Here, we perform multivariate representational analysis and show category formation as a shift from original categories grouped by visual features to categories grouped by use. Lastly, we wish to emphasise that our research is highly relevant not only to the visual learning/expertise community but also to anyone interested in BMI/BCI, motor control, tool use, body augmentation and body representation.

Major points.

2.1. In the discussion I think the authors oversell their results as evidence against the embodiment hypothesis. The authors do discuss some limitations of their methods and results but I still think they go too far in their conclusions. The present study does not investigate fronto-parietal-cerebellar regions during actual prothesis use so the embodiment theory has not been directly tested in my opinion. To me it seems reasonable that the OTC in expert prothesis-users should be better at differentiating between prosthetic limbs and real hands compared to controls. But does this really matter for how visual information about the prosthesis is processed by parietal, frontal and cerebellar regions during prosthesis use? Take hand-held tools for example. Clearly the OTC can differentiate body parts from tools but tools can be embodied in the sense that some of the same sensorimotor mechanisms used to control real limbs are re-used for effective control of hand-held tools. So, my
point is simply that I think the authors should have a more balanced discussion with respect to the significance of the present results for theories of prothesis embodiment.

We are grateful for this opportunity to improve our discussion. We fully agree with the reviewer that the lack of visual embodiment does not entail that the prosthesis cannot be embodied at another level/brain areas.

Discussion (1^st^ paragraph):

“*Despite benefiting from hand-selective cortical resources [29], the prosthesis is not ‘embodied’ into people’s visual hand representation. However, since the experimental design and analysis we implemented were a priori focused on visual hand embodiment in OTC, it is possible that other brain areas might find a different representational ‘solution’ to support the daily use of an artificial limb.”*

Discussion (3^rd^ paragraph):

*“One potential solution originates from the fact that embodiment is a multifaced phenomenon [17], with distinct levels (i.e. phenomenological – does the prosthesis feel like my hand? cognitive – do I react to/with the prosthesis like I would to/with my own hand? ; neural – is the prosthesis represented via the same mechanisms as a hand? [7]). In another recent study where we probed the phenomenological and cognitive levels of prosthesis embodiment, we found it to correlate with prosthesis usage [28]. Here we focused our research on the neural level, since it is entirely unknown whether objective representation of the prosthesis as a body-part associates with prosthesis acceptance and usage, let alone benefits it. It is possible, and even likely, that embodiment manifests differently in each of these distinct levels, which may also vary in their importance or even relevance for successful technological implementation [7]”*

We have now clarified throughout the manuscript that we are probing visual embodiment, and not sensorimotor embodiment. We also elaborate on the multiple conceptual reasons for studying visual embodiment in general and visual embodiment of prosthetic limbs within the EBA specifically. These are now included more clearly in the introduction and discussion.

First and foremost, embodiment is a multisensory experience, which is predominantly measured by means of visual manipulations. An interesting case in this context is the rubber hand illusion (RHI), which is often taken as the paradigmatic example of embodiment. And yet some have argued that the rubber hand is not embodied at the sensorimotor level, but rather at the visual level (de Vignemont, 2018; Kammers, Longo, Tsakiris, Dijkerman, & Haggard, 2009). Furthermore, Tsakiris (2010) have argued that the key difference between the rubber hand and a tool, and the reason for which one cannot have ownership for a tool, is because of the lack of visual embodiment. Visual embodiment is thus conceived by some as a gateway for the sense of bodily ownership. The relationship between prosthesis embodiment and tool embodiment is a fascinating one, unfortunately considering the scope of the current study we feel like we will not be able to cover it with all its complexity. We have, however, followed up on this study with an experiment specifically designed to address this topic, whether the categorisation effect observed here is specific for prosthetics, that are designed to substitute a body function, or are a general feature of expert tool-use.

Moreover, prosthesis usage is highly visually-guided (Antfolk et al., 2013). As such, research of prosthesis embodiment should build on the importance of visual experience in daily prosthesis usage. An additional, more minor, consideration for us was that sensorimotor embodiment would be difficult to test since in congenital one-handers, the sensorimotor homunculus is architecturally changed compared with controls (Hahamy et al., 2017; Hahamy & Makin, 2019; Wesselink et al., 2019). Therefore, differences observed in these areas would be difficult to interpret.

Introduction (2^nd^ paragraph):

*“Embodiment is an umbrella term for the multisensory association of external objects with body representation, which engages multiple levels from both conceptual and perceptual perspectives (see Discussion). From a neural standpoint, embodiment is defined by the successful allocation of brain resources, originally devoted to controlling one’s own body, to represent and operate external objects [17]. Visual embodiment is particularly relevant for studying prosthesis representation. This is because prosthesis usage is highly visually guided [18], and because visual embodiment has been suggested as a gateway for the sense of bodily ownership [19], a desired feature of prosthesis usage [9,10]. Previous efforts to test visual prosthesis embodiment have been centred on an illusion of body ownership over the prosthesis (most commonly, the rubber hand illusion, which relies on visual manipulations), with mixed success [11,20–23].”*

EBA is a body- and hand-selective region, and is therefore suitable to testing embodiment (i.e. hand-like representation of artificial limbs). We (Orlov, Makin, & Zohary, 2010) and others (Bracci, Caramazza, & Peelen, 2015) have shown that this area selectively responds to body parts, including hands. EBA is not strictly visual, as it also contains sensorimotor attributes. We and others have shown that the region responds to hand movements (Astafiev, Stanley, Shulman, & Corbetta, 2004; Orlov, Porat, Makin, & Zohary, 2014) and passive touch (Tal, Geva, & Amedi, 2016). This is also very clearly demonstrated in blinds (Wesselink, Kikkert, Bridge, & Makin, 2018). EBA’s connectome further associates it with hand actions: it is uniquely connected to the sensorimotor hand area (Tal et al., 2016). We have recently shown that inter-modal connectivity between visual and motor hand-selective regions is modulated by the degree to which individuals use their prosthetic limb (Van Den Heiligenberg et al., 2018). This evidence indicates that this region is sensitive to daily prosthesis usage. Finally, and of particular relevance to our key research question, there is a known overlap between the hand-selective and tool-selective regions within the extrastriate area, making it an ideal brain area to test for the hypothesis that a prosthesis is represented like a tool (Bracci, Cavina-Pratesi, Ietswaart, Caramazza, & Peelen, 2012).

Introduction (3^rd^ paragraph):

*“The OTC is known to contain distinct visual representations of different object categories [30], for example: hands [31,32], tools [33], faces [34], and bodies [35]. It was previously shown to contain overlapping visual and motor body-part selective representations [31,36], and even responds to touch [37,38]. OTC’s connectome associates it with hand actions. For example, hand and body-selective visual areas uniquely connect to the primary sensorimotor hand area [37]. These characteristics qualify the OTC as an ideal candidate for studying action-related visual body-representation. We previously found that individuals who used their prosthesis more in daily lives also showed greater activity in OTC’s hand-selective visual areas when viewing images of prostheses, and greater functional coupling with sensorimotor hand areas [29]. This result demonstrates that prosthesis users are able to engage visuomotor hand-selective areas while (passively) representing a prosthesis.“*

Introduction (3^rd^ paragraph):

*“Alternatively, since the visual hand-selective regions partially overlap with hand-held tool representation [39], the observed activity gains may reflect the prosthesis being represented as a tool.“*

While our a priori focus was on OTC (and EBA in particular), following the reviewers comments we have proceeded with some exploratory analysis in parietal cortex.

For these reasons, we believe this region provides a key model for addressing our question, and have thus designed the experiment to activate this region (i.e. static images). However, in the revised manuscript we also explored a few other brain regions. First, we include an additional analysis with similar finding, focused on an independent hand-selective ROI in OTC. This additional exploratory analysis demonstrates that our results are not specific to the chosen ROI (See supplementary results). Second, since the group level analysis showed significant activity in the intra parietal sulcus (IPS), in the revised manuscript we include additional exploratory analysis performed in IPS, as detailed below.

Results (5th section):

*“****Prosthesis representation beyond EBA***

*To demonstrate that our results are not specific to our ROI definition of EBA, we’ve repeated the same analysis, and found similar results, in ‘hand’ and ‘tool’ ROIs within OTC, generated from the meta-analysis tool Neurosynth [47] (see Supplementary results and Figure S4).*

*The stimuli used in the current study were specifically designed to interrogate the information content underlying prosthesis representation in body-selective regions in the OTC. Nevertheless, as demonstrated in Figure S5A, the visual stimuli also significantly activated the intraparietal sulcus (IPS), providing us with an opportunity to explore visual prosthesis representation in a dissociated brain area (though notably, this was observed less consistently within individual subjects). Since 11 of the participants did not have enough significantly activated voxels within IPS to meet our ROI criteria, we constructed an anatomical ROI based on the Juliech Histological Atlas in FSL, including human intraparietal (hIP)1-3 bilaterally [48,49] (Fig. S5B). With respect to RSA, we did not find significant differences between prosthesis users and controls when comparing both the hand- and prosthesis-similarity indices, even when wavering corrections for multiple comparisons which are customary for exploratory analysis (hand-similarity: t(54)=0.71, p=0.48; prosthesis-similarity: t(54)=-0.45, p=0.66). However, we did find that users showed significantly greater dissimilarity (greater distances), relative to controls, when comparing the representation of their own prosthesis to that of both hands and tools (hand: t(25)=10.11, p<0.001; tool: t(25)=4.62, p<0.001, corrected for 2 comparisons, see Fig. S5C). This analysis indicates that similar to the OTC, in parietal cortex too, users’ own prostheses are less similar to hands and tools, providing support in favour of the categorisation interpretation, and against the embodiment hypothesis. “*

Supplementary figure:

*“****Supplementary Figure S5. Intraparietal Sulcus (IPS) analysis.*** *(A) Univariate activations in prosthesis users. Results of the group level univariate contrast of (Active Prosthesis + Cosmetic Prosthesis) > Objects show that at the group level the IPS is also activated. (B) The IPS region of interest was taken from the Juelich Histological Atlas (30% probability of hIP1, hIP2, and hIP3). (C) Hand (left) and tool (right) distances from users’ ‘own’ prosthesis in IPS. Individual distances were normalised by the controls’ group mean distance, depending on the visual features of the ‘own’ prosthesis (hand-likeness). A value of one indicates similar hand/tool distance to controls. Users showed significantly greater distances between their own prosthesis and hands (t(25)=10.11, p<0.001contrary to the embodiment hypothesis. A significant increase in the distance of the ‘own’ prosthesis from tools was also observed (t(25)=4.62, p<0.001).”*

2.2. The authors do discuss some limitations of the study with respect to the embodiment question, most notably the use of 2-D pictures of hands rather than real protheses and the focus on a single visual area rather than a network of sensorimotor regions. This is all very good, but to me a further limitation is that the authors used hands and prostheses depicted from different visual orientations, and most problematically, mixing up first person perspective and third person perspective. Does this not cause a problem for the interpretation of the results? Hands (or protheses) of strangers viewed from a third person perspective should not be embodied but simply treated as external visual object. How could this mixing of perspectives have influenced the current results?

We apologise for not clarifying this important consideration. A key to our approach was the ability to compare representations across prosthesis users (congenital and acquired one-handers) and between prosthesis users and controls. To balance between the groups, it was crucial for our design to use multiple perspectives of both hands and prostheses. Note that this design aspect is highly consistent with our own previous cognitive research showing adaptable categorisation of hands and tools in prosthesis users (van den Heiligenberg, Yeung, Brugger, Culham, & Makin, 2017), as well as other’s research on visual hand representation in amelia patients (Striem-Amit, Vannuscorps, & Caramazza, 2017).

To our knowledge, currently there is no clear evidence that OTC/EBA represents allocentric and egocentric perspectives differently. There is previous evidence to suggest that the right EBA is more strongly activated by allocentric versus egocentric images (Saxe, Jamal, & Powell, 2006), or for self vs other body-part images (Myers & Sowden, 2008). But these reported effects were modest, and not well replicated (Hodzic, Muckli, Singer, & Stirn, 2009). Regardless, since in our study we identify similar effects across hemispheres, we don’t believe we are impacted by these potential differences. We now convey this additional information in the revised manuscript.

Methods (Stimuli section, 2^nd^ paragraph):

*“We chose to use multiple configurations of hands and prostheses to probe the experience of congenital one-handers and control participants (who mostly see prostheses/hands-of-the-missing-side from a 3rd person perspective, respectively). We note that previous studies probing visual hand representation in similar populations [82], including specifically in OTC [54] used a similar approach. We further note that the few studies finding differences between egocentric/allocentric [84] or self/others [85] visual hand representation in OTC identified lateralised effects to the right OTC, whereas our effects are comparable across hemispheres.”*

Discussion (4^th^ paragraph):

*“The use of generic hand images that are not the participants’ own hands can also be limiting when approaching questions of embodiment. Here it is important to mention that despite using a profoundly non-ecological task in the scanner, the resulting brain representations, as captured with our prosthesis-similarity index, correlated significantly with the extent of everyday prosthesis usage. Therefore, despite the inherent limitations of our fMRI task, our task outcomes are ecologically relevant. Still, it is possible that other brain areas involved more directly in motor planning would produce other representational structures with respect to hand representation.”*

2.3. I think the authors needs to work more on the last paragraph of the discussion. I see two problems with this “conclusion paragraph”. The first is that the authors suddenly introduce a new third interpretation of their data (“appendage represented as a body part”). This seems out-of-place here in the conclusion paragraph. If this is an important alternative interpretation – which I think it is – then it should be discussed more extensively earlier in the discussion paragraph. Also, the authors should more explicitly state how this “appendage interpretation” relates to the two hypotheses outlined in the introduction and how it fits with the results more precisely. But if this idea is merely a speculation it might be better to drop it all together. The second problem I had with the “conclusion paragraph” is that the authors argue against using rubber hand illusion as a tool for prosthesis embodiment research. I do not follow. The present study did not investigate the rubber hand illusion or the sense of ownership of prosthetic limbs, so to me this part reads like an unnecessary speculative overstatement. In sum, I recommend a shorter conclusion-paragraph without speculations or introduction of new concepts and interpretations.

First, we apologise for the misunderstanding. Our conclusion was elaborated in the first sentence only. We did not intend this as a concluding paragraph (beyond the first sentence), but instead an opportunity to communicate a more speculative perspective on our research, as stated in the original manuscript. We have now restructured our discussion to avoid this confusion. This alternative interpretation indicates that we should expand our repertoire of experimental paradigms while studying embodiment from the hand to the body (either as a whole, or using other sub-categories, such as appendages). Since we are not interpreting, but rather speculating, we don’t think it should be considered as an over-interpretation. This speculation is meant to help the community think a little outside its box and to stimulate new discussion. As such, we think it is valuable. To avoid further confusion, we have moved this alternative interpretation as one of three, where we try to balance our discussion of our findings in the greater realm of embodiment.

Discussion (5^th^ paragraph):

*“Thirdly, and most speculatively, it is possible that the prosthesis may still be represented in OTC as a body part, but one that is not a hand. After all, prosthesis users have strong semantic and sensorimotor knowledge of a hand (all users in the study had one intact hand; acquired amputees had prior experience of their missing hand, including lingering phantom sensations; see also [54] for related findings showing normal visual hand representation in individuals born without both hands). Their experience with operation of a prosthesis is fundamentally different from that of a hand. If body representation is not strictly tuned to the specific fine-grained features of a given body part (e.g. the digits of the hand), but is instead also represented at a higher level (e.g. effectors [55] or based on other functionality features [56]), then the dissociation of prostheses from hand representation observed in our study should not be taken as evidence for lack of embodiment per se, but rather lack of embodiment as a hand. Therefore, we propose that future studies of artificial limb embodiment should not be limited to identifying and/or quantifying hand representation (as the current common practice, e.g. using the rubber hand illusion).”*

We also note that we did intent to look for a relationship between prostheses and a body part that is not a hand in our original design (which was our motivation to include lower limbs in the experimental design). But upon analysis we found that the lower limbs were equally distant from the other conditions of interest (hands, cosmetic prostheses and active prostheses) for both groups. We believe that this is to do with the fact that lower limbs are represented at a distinct (potentially orthogonal) dimension from that of prostheses, hands and tools. As such, these might not be suitable for measuring modulation in representational distances reported in this manuscript.

Methods (Stimuli section, 1^st^ paragraph):

*“Additional conditions which were also included in the fMRI paradigm and initial analysis but not reported here were: dismorphed images (originally intended to account for low-level visual activity but discarded after univariate analysis revealed increased activity in OTC), and lower limbs (intended as a control body part but not included in final analysis).”*

Minor points

2.4. In my opinion, the statement “In other words, prosthesis users do not, on average, ‘embody’ prostheses as hands more than controls.” is an overstatement. This conclusion is based on a non-significant result from a frequentist statistical test. In other words, all you can say is that you did not find significant evidence against the null hypothesis. But this does mean that you can conclude that the null hypothesis is true. Please tone down this statement or present results from a Bayesian statistical analysis that support the null hypothesis. (This comment refers to a sentence in the results paragraph with the heading “Prosthesis-like (categorical), and not hand-like (embodied) representation of prostheses in prosthesis users”)

We fully take this point and have now removed this sentence. We also added a Bayesian analysis, which confirmed that prosthesis users did not show greater similarity to a visual hand representation. This is now described in the manuscript as follows:

Results (2^nd^ Section, 1^st^ paragraph):

*“A Bayesian t-test provided substantial evidence in support of the null hypothesis (BF=0.2), i.e. that on average amputees do not represent an unfamiliar prosthesis more similarly to a hand than controls.”*

Methods (Statistical Analysis):

*“All statistical testing was performed using SPSS (v24), with the exception of the Bayesian analysis which was run on JASP [99]). … To interpret key null results, we ran a one-tailed t-test. The Cauchy prior width was set at 0.707 (default). We interpreted the test based on the well accepted criterion of Bayes factor smaller than 1/3 [100,101] as supporting the null hypothesis.”*

2.5. In the methods section (under “Stimuli”) please add a motivation for mixing the presentations of the pictures of the hands and protheses from the first- and third-person perspectives (also see point 2 above). Please also clarify which perspective that was used for the own hand stimuli. Finally, add an explanation for why the shoe condition was not included in the analysis.

Methods(Stimuli section, 2^nd^ paragraph):

*“… each of the three prosthesis conditions involved multiple shots of a single prosthesis of different orientations. We chose to use multiple configurations of hands and prostheses to probe the experience of congenital one-handers and control participants (who mostly see prostheses/hands-of-the-missing-side from a 3rd person perspective, respectively). “*

Methods(Stimuli section, 3^rd^ paragraph):

*“Shoes were selected as a familiar external object that was intended to exert similar cognitive effects (e.g. in terms of arousal) as the prosthesis, and therefore minimize differences in the scan time course across groups. Since we had no a priori interest in studying shoe representation, the shoe condition was not included in further analysis.”*

2.6. It would have been interesting to have ratings regarding how much the amputees embodied their prothesis in every-day life. I assume that according to the embodiment hypothesis this should have some relationship to the neural representation of protheses. If I am not misinformed, most prothesis users describe their prothesis more like a tool than an actual hand.

While we did not include embodiment questionnaires in the present study cohort, we have administered them in a further study, involving 44 prosthesis users (of which 23 with congenital hand loss). As a whole, one-handers tended to mildly agree with embodiment statements (0.47 on a +3 to -3 scale) although this wasn’t significantly different than 0 (i.e. they responded neutrally). Of relevance to the reviewer’s suggestion, they did not respond more positively to the statement ‘it seems like the prosthesis is a part of my body’ than they responded negatively to the statement ‘it feels like my prosthesis is a foreign body’ (Wilcoxon Signed-rank test, the sign for the latter answer was reversed for the purpose of the test; Z = -0.936, p=0.36). Further details of this study will become shortly available on Biorxiv, and we plan to make it available prior to the publication of the present study.

Introduction (2^nd^ paragraph):

*“We have recently found that as a whole, one-handed individuals tend to respond neutrally to embodiment statements in relation to their own prostheses [28]. Moreover, these measures of embodiment tend to rely on an explicit sense of body ownership, which might not be a necessary consequence of implicit neuronal embodiment (i.e. reallocation of body-part resources to represent or control the prosthesis [17].“*

Discussion (3^rd^ paragraph):

*“In another recent study where we probed the phenomenological and cognitive levels of prosthesis embodiment, we found it to correlate with prosthesis usage [28]. Here we focused our research on the neural level, since it is entirely unknown whether objective representation of the prosthesis as a body-part associates with prosthesis acceptance and usage, let alone benefits it.”*

2.7. In the second discussion paragraph you mention the sensorimotor related regions in the frontal and parietal regions, but give no references. Please add a couple of references here.

We’ve added a reference to the mentioned sentence:

*“For example, our use of static images was designed to drive activity in the OTC, but not in other sensorimotor-related regions in the parietal and frontal regions [53], thereby limiting our conclusions to high-level visual cortex.”*

This is based on a collaboration study we ran with Jody Culham examining activity associated with static images, versus, videos of hands and tools. Unfortunately, this evidence hasn’t been published yet, but these have been presented in VSS in a published abstract which we now cite.

2.8. I miss page numbers in the manuscript.

This was intentional, to avoid conflicts with the PDF page numbers. We have now added page numbers to our Word doc.

Reviewer #3: The authors present an interesting study aiming at characterising how the visual cortex, and more specifically the extra striate body area, reacts to images of prostheses, tools and hand in prosthesis users and controls. The topic and objective of the paper, understanding how the brain embodies technologies -here a prosthetic hand-, is not only important conceptually but also has major implication for rehabilitation since technological development is not sufficient for successful device adoption and usage. Indeed, to better understand how amputees adopt their prosthesis, one needs to measure objectively embodiment. The authors decided to have an implicit measure of embodiment based on recording the activity of region in the visual system known to typically show specific responses to tools and body-parts like hands. This study is an important extension of a previously published paper showing that individuals who used their prosthesis more in daily lives also
showed greater activity in the OTC’s hand-selective visual areas when viewing images of prostheses (van den Heiligenberg, Brain, 2018). The main claims of the paper is straightforward: in contrast to what is predicted to an embodied view of brain (re)organization in amputees, the authors show that the upper limb prothesis is represented in the occipital cortex differently that the hands. The paper is clearly written and the study was elegantly designed and expertly executed. Authors should be praised  to rely on fMRI data of 32 individuals missing a hand, which is a sample size larger than typically reported in the literature. Here follows some suggestion that may help to improve the study.

3.1 The authors set out to explore two alternative hypotheses (embodiment vs categorisation hypotheses) by mainly contrasting brain activity evoked by prostheses versus hand. However an important third hypothesis is not fully tested in my view: that prostheses are represented not like hand but like tools. This third hypothesis presume that prostheses representations will anchor on an existing category (tools) which is different from what the authors suggest, namely the creation of a new categorical distinction due to new conceptual (visual? -see below) knowledge.

Indeed, this third hypothesis is embedded in our embodiment hypothesis and index. In short, we devised the embodiment index as the relative distance of the prosthesis to the hand, while considering its distance from the tool. As such, a negative embodiment effect actually indicates increased association with a tool. Since our statistical tests are two-tailed, this possibility has been implicitly tested, with non-significant results. This has now been further clarified in the text:

Introduction (4^th^ paragraph):

*“Two main hypotheses were tested: the embodiment hypothesis, assessing whether prosthetic limbs are in fact represented visually as hands and not tools; and, the categorisation hypothesis, assessing whether a new ‘prosthesis’ category has formed. To provide distinct predictions for each of these hypotheses, we studied representational similarities between hand, tools, and upper limb prosthetics images (both cosmetic - designed to resemble hand appearances; and active – designed to afford a grip, e.g. a “hook”; Figure 1A) and compared prosthesis users to controls. Broadly speaking, the visual hand embodiment hypothesis predicts that compared to controls, the various prosthesis conditions in prosthesis-users will be more similar to hands than to tools (notice that this prediction also allows us to test the inverse prediction – that prostheses are represented more like tools in one-handers). The categorisation hypothesis predicts that in prosthesis-users, the prosthesis conditions will be more similar to each other relative to hands and tools.”*

Results (2^nd^ section, 1^st^ paragraph):

*“… This hypothesis predicts that in prosthesis-users, each of the two prosthesis conditions will be more similar (smaller distance) to hands than to tools, compared to controls (quantified as a hand-similarity index, see Methods). Notice that the hand-similarity index is the inverse of a tool-similarity index. Therefore, significantly negative embodiment should be taken as evidence for association of the prosthesis with a tool.”*

*“When comparing hand-similarity indices based on the multidimensional distances across participant groups, we found no significant differences (t(54) = 0.47, p=0.64; Figure 3A). A Bayesian t-test provided substantial evidence in support of the null hypothesis (BF=0.2), i.e. that on average amputees do not represent an unfamiliar prosthesis more similarly to a hand than controls.”*

Methods (Hand-Similarity and Prosthesis-Similarity Indices):

*“For instance, the embodiment hypothesis predicts that for both prostheses (active and cosmetic) the distance from tools would grow (will be less similar), while the distance from hands will shrink (will be more similar). Therefore, the formula used to calculate the Hand-Similarity index was: (Tools↔CosmP + Tools↔ActP) – (Hands↔CosmP + Hands↔ActP), where ↔ indicates the distance between a pair of conditions.*

3.2 As discussed by the authors, the main finding (as summarised above) could well be explained by visual expertise. Alternatively, since it was shown that body-selective regions also link to the parietal cortex to represent body and tool actions, one may think that the organisation of this brain region also depends on the use of the prosthesis. At the moment the paper does not disentangle these two alternatives which would tell us important information about the mechanisms underlying the brain reorganisation in amputees and people born without hand. Would it be possible to find a strategy to disantagle those two alternatives by analysing further the brain representation of your own prosthesis versus another prosthesis with similar design-function? In this vein, it might also be interesting to scan the brain of people involved in rehabilitative training of amputees since one can assume similar visual expertise without the motor component of use. I think also that this topic
(visual vs user-dependent expertise) should be put upfront in the paper in the introduction.

We absolutely agree with the reviewer that this is a really interesting conceptual question. But from a real-life perspective, one cannot acquire motor experience with a prosthetic arm without concurrent visual experience. In fact, as we highlight in the manuscript, prosthesis usage is notoriously over-reliant on visual feedback. Therefore, for our model (prosthesis usage), visual and motor expertise are inherently linked and as such we did not provide additional controls to dissociate them. Based on this comment, as exploratory means we took advantage of some limited visual experience in the control group, which included prosthetics professionals, as well as friends and family of the users. We note that only a subset of the group (n=14) had experience in viewing prosthesis users, and that even so the level of visual expertise for observing a prosthesis being used is likely marginal compared to actual usage. In other words, the visual expertise we could measure in the controls is not matched to the visuomotor experience of prosthesis users. But the varying levels of visual experience in this subset of controls helps us consider the role of visual familiarity, though with limited statistical power. We found no correlation between the prosthesis usage observation score and the prosthesis similarity index, r(12)=0.07 p=0.805. This result potentially hints that visual familiarity alone is not sufficient to drive the categorisation effect. But considering the limited sample size and the null result we decide not to include this analysis in the manuscript. Indeed, an interesting follow-up to our study could be recruiting people highly involved in rehabilitative training of amputees to disentangle visual vs. use-dependent expertise. Finally, with regards to the comment on the own prosthesis analysis. What the reviewer is suggesting (comparing the representation of the own prosthesis to a representation of a similar prosthetic in controls) is actually very similar to the analysis we’ve conducted.

Introduction (3^rd^ paragraph):

*“As a third option, since object categorisation in OTC is thought to be based on their semantic and functional properties[40], expert prosthesis usage may result in the emergence of prostheses representation as a new ‘category’, diverging from its existing natural categories (e.g. ‘hands’, ‘tools’). This third alternative is consistent with recent evidence showing that visual expertise can contribute to the shaping of categorical representation in OTC [41] (see Discussion).”*

Methods (Own prosthesis representation):

*“Our approach was designed to overcome two challenges: First, controls do not have an ‘own’ prosthesis, and; second, the visual and operational features of different prostheses may vary significantly, and need to be accounted for before similarity measures can be averaged across all prosthesis users. Therefore, for each participant, we normalised (divided) the ‘own’ prosthesis distance by the mean distance of a similarly-looking/operating prosthesis, as found in controls.”*

3.3 Authors focused their study on body selective regions. Why not conducting similar analyses on more specific ROIs selective to hands or tools? I understand that body selective regions could be defined independently (univariate analyses) from the conditions of interest undergoing multivariate analyses, which may not be the case for hands/tools. I am however wondering if the author could use a data split-half strategy (or any type of crossvaligdation technique) or relying on hand and tool related ROIs from the literature to achieve this goal?

We focused on EBA because we wanted an ROI definition which is independent of the ‘hand’ and ‘tool’ categories. Following the reviewer’s suggestion, we’ve extracted a ‘hand’ and a ‘tool’ ROI from Neurosynth and received similar results to those found in our EBA ROI. We have now included this analysis in the supplementary section an refer to it from the main text:

Results (5^th^ section):

*“To demonstrate that our results are not specific to our ROI definition of EBA, we’ve repeated the same analysis, and found similar results, in ‘hand’ and ‘tool’ ROIs within OTC, generated from the meta-analysis tool Neurosynth [47] (see Supplementary results and Figure S4).”*

Supplementary Results:

“*To demonstrate that our results are not specific to the ROI definition used here for EBA, we’ve conducted the same analysis in ‘hand’ and ‘tool’ ROIs generated from the meta-analysis tool Neurosynth [1] (https://neurosynth.org/analyses/terms/hand/, https://neurosynth.org/analyses/terms/tools/). Using the association maps for the words: ‘hand’ and ‘tools’, ROIs were defined by using all significant voxels within the OTC (see Methods for definition of OTC). It is important to note that there is some partial overlap between the ‘hand’ and ‘tools’ ROIs, particularly in the left hemisphere (see figure S4). This is a known limitation of the spatial distribution of hand- and tool-selectivity in OTC, which is the primary reason we opted for an a priori independent ROI definition of EBA. Furthermore, as these ROIs were transformed from standard space and were not localised for each individual subject, the probabilistic ROI approach suffers from potential for reduced statistical power.*

*Both the ‘hand’ and ‘tool’ ROIs showed no significant visual hand-similarity (embodiment) group differences or correlation with prosthesis usage (p>0.2 for all). The ‘hand’ ROI showed a significant prosthesis-similarity (categorisation) group difference (t(54)=-2.84, p=0.01), with prosthesis users showing a stronger categorisation effect, and a trend toward significance for the correlation with prosthesis usage (r(30)=0.32, p=0.08). The ‘tools’ ROI did not show a significant categorisation prosthesis-similarity group effect (t(54)=-0.56, p=0.58), but showed a significant correlation with usage (r(30)=0.40, p=0.03).*

*Furthermore, when performing the ‘own prosthesis’ analysis, the Neurosynth ‘hand’ ROI showed a significant shift of the own prosthesis away from hands (t(25)=6.70, p<0.001) but not away from tools (t(25)=1.78, p=0.087). Neurosynth’s ‘tool’ ROI showed a significant shift of the own prosthesis away from both hands (t(25)=4.19, p<0.001) and tools (t(25)=4.3, p<0.001). This additional analysis confirms that our key finding in OTC are relatively robust.”*

***Supplementary Figure S4. Neurosynth ‘hand’ and ‘tool’ ROIs.*** *Using the association maps for the words: ‘hand’ (blue) and ‘tools’ (green), ROIs were defined by using all significant voxels within the OTC. These ROIs are projected on inflated brain for visualisation****.***

3.4 In a previous study, the authors found that individuals who used their prosthesis more in daily lives also showed greater activity in the OTC’s hand-selective visual areas when viewing images of prostheses (van den Heiligenberg, Brain, 2018). Here, they find that when comparing activity levels between controls and prosthesis- users within this region, no significant differences were found for each of the image conditions of hands, tools and prostheses . How do these two observations made on the same dataset fit?

We would like to highlight that the results are not identical. This is because in the Brain paper we focused on hand-selective regions whereas here we are using an EBA ROI, to avoid circularity in our analysis. Following the reviewers comments we repeated the analysis from the Brain paper comparing prosthesis users vs. controls, we used man-made objects as a control condition, considering it can broadly activate the OTC but is irrelevant for our research question. We find greater activity in prosthesis users than controls for functional prostheses (t=-2.18, p=0.03).

3.5 A group comparison of the hand-similarity index between controls and prosthesis users showed no significant differences. In addition, users showed significantly greater distances between their own prosthesis and hands. Both results are thought to contrast with the embodiment hypothesis but the link between those two results is unclear to me.

We apologise for causing this confusion. The key difference between these two measures is that the hand-similarity index measures other people’s (unfamiliar) prosthesis representation.

Under the embodiment hypothesis the representation of prostheses (both user’s own and other’s) should become more similar to hands and less similar to tools. The hand-similarity index quantifies how much the representation of other’s prostheses is more similar to hands than tools. The fact that there is no difference between controls and prosthesis users in this measurement teaches us that prosthesis usage does not result in more hand-like representation for unfamiliar prostheses. We have now included a Bayesian analysis as well to support this null result.

But we also wanted to study the users’ representation of their own familiar prosthesis. Here we needed to account for the visual and operational features of the prostheses type, which we know from the control participants can impact the prosthesis distance to hands. We therefore normalised the hand-‘own’ distance by control’s distances to hands, per prosthesis type. Greater distance found between users’ own prosthesis and hands indicates less similarity between the representations of the two conditions, after accounting for the prosthesis type. Since the prosthesis is represented further away from hands compared to controls, this analysis, which we have now also replicated in the parietal cortex, provided direct negative evidence for the embodiment theory.

We have now adapted the text throughout the results and discussion to better reflect these differences:

Results (2^nd^ section, 1^st^ paragraph):

*“When comparing hand-similarity indices based on the multidimensional distances across participant groups, we found no significant differences (t(54) = 0.47, p=0.64; Figure 3A). A Bayesian t-test provided substantial evidence in support of the null hypothesis (BF=0.2), i.e. that on average amputees do not represent an unfamiliar prosthesis more similarly to a hand than controls.”*

Results (2^nd^ section, 5^th^ paragraph):

*“Comparing the correlation between prosthesis usage and the indices for the two hypotheses (embodiment and categorical) revealed a significant difference (z=-2.52, p=0.01). Therefore, at least when considering unfamiliar prostheses, the prosthesis-similarity index is a better predictor of prosthesis usage than the hand-similarity index.“*

Results (4^th^ section, 2^nd^ paragraph):

*“Based on the embodiment hypothesis, users should show greater similarity between their own prosthesis and hands. Instead, we found that users showed significantly greater dissimilarity (greater distances), relative to controls, indicated by a normalised distance which was greater than 1 [t(25)=2.85, p=0.009]. This analysis therefore shows that users’ own prostheses are less similar to hands, providing direct evidence against the embodiment hypothesis.”*

Discussion (1st paragraph):

*“When comparing the two models’ success in explaining inter-individual differences between prosthesis users, we find that the prosthesis category model was significantly more correlated with prosthesis usage. This indicates that for visual representation of unfamiliar prostheses, categorisation provides a better conceptual framework than embodiment. Together with preliminary results showing that prosthesis users exhibited a less hand-like representation of their own prosthesis in parietal cortex, our results collectively show that neural visual embodiment does not predict successful adoption of wearable technology by the human brain.”*

3.6 The prosthesis categorisation effect was found in both congenital and acquired one-handers suggesting no "sensitive period" for this effect to emerge. However the analyses in link to the comparison of these two groups could be further developed in the manuscript.

We apologise for not making this important point clearer. We did compare the two groups for the categorisation similarity index in the original manuscript, using an ANCOVA with sub-group (congenital vs acquired) as a fixed-effect and usage as a covariate.

Results (2^nd^ section, 4^th^ paragraph):

*“The ANCOVA revealed no significant sub-group effect (F(1,29)=2.02, p=0.17) – demonstrating that categorisation does not significantly differ due to cause/developmental period of hand-loss; and a nearly significant usage effect (F(1,29)=4.11, p=0.052) – indicating that the relationship between categorisation and usage is relatively independent from potential sub-group effects.”*

We did not explore group differences for the hand-similarity index because it did not yield significant results, and for the distance between ‘own’ prosthesis and hands because of the reduced sample size. Following the reviewer’s query, we have now explored these two additional results for group differences. We found no significant sub-group effect for the hand-similarity index (t(30)=-0.615,p=0.54), and no significant sub-group effect for the distance between own prosthesis and hands (t(24)=0.1, p=0.92). As such, to date we have not identified any significant differences between amputees and congenital one-handers with respect to visual prosthesis representation.

3.7 The analysis were implemented a priori focusing on prosthesis and hand representation in the occipital cortex. Do the authors have speculation about what is happening in other brain regions like the sensorimotor cortex which is thought to also activate in link to action related objects- it is possible that a different representational structure support the use of an artificial limb in those regions?

We fully agree with the reviewer that other brain areas might show a different representational structure to support artificial limb use. This is option is now more pronounced in our discussion:

Discussion (1^st^ paragraph):

“*Despite benefiting from hand-selective cortical resources [29], the prosthesis is not ‘embodied’ into people’s visual hand representation. However, since the experimental design and analysis we implemented were a priori focused on visual hand embodiment in OTC, it is possible that other brain areas might find a different representational ‘solution’ to support the daily use of an artificial limb.”*

Discussion (4^th^ paragraph):

*“… Still, it is possible that other brain areas involved more directly in motor planning would produce other representational structures with respect to hand representation. Future research aimed at this question should take into consideration that at present, commercially available prosthesis motor control is fundamentally different from that of motor control of the hand, producing further potential challenges for sensorimotor embodiment”*

In the revised manuscript we also explored a few other brain regions. First, we include an additional analysis with similar finding, focused on an independent hand-selective ROI in OTC. This additional exploratory analysis demonstrates that our results are not specific to the chosen ROI (See supplementary results). Second, since the group level analysis showed significant activity in the intra parietal sulcus (IPS), in the revised manuscript we include additional exploratory analysis performed in IPS, as detailed below.

Results (5th section):

*“****Prosthesis representation beyond EBA***

*To demonstrate that our results are not specific to our ROI definition of EBA, we’ve repeated the same analysis, and found similar results, in ‘hand’ and ‘tool’ ROIs within OTC, generated from the meta-analysis tool Neurosynth [47] (see Supplementary results and Figure S4).*

*The stimuli used in the current study were specifically designed to interrogate the information content underlying prosthesis representation in body-selective regions in the OTC. Nevertheless, as demonstrated in Figure S5A, the visual stimuli also significantly activated the intraparietal sulcus (IPS), providing us with an opportunity to explore visual prosthesis representation in a dissociated brain area (though notably, this was observed less consistently within individual subjects). Since 11 of the participants did not have enough significantly activated voxels within IPS to meet our ROI criteria, we constructed an anatomical ROI based on the Juliech Histological Atlas in FSL, including human intraparietal (hIP)1-3 bilaterally [48,49] (Fig. S5B). With respect to RSA, we did not find significant differences between prosthesis users and controls when comparing both the hand- and prosthesis-similarity indices, even when wavering corrections for multiple comparisons which are customary for exploratory analysis (hand-similarity: t(54)=0.71, p=0.48; prosthesis-similarity: t(54)=-0.45, p=0.66). However, we did find that users showed significantly greater dissimilarity (greater distances), relative to controls, when comparing the representation of their own prosthesis to that of both hands and tools (hand: t(25)=10.11, p<0.001; tool: t(25)=4.62, p<0.001, corrected for 2 comparisons, see Fig. S5C). This analysis indicates that similar to the OTC, in parietal cortex too, users’ own prostheses are less similar to hands and tools, providing support in favour of the categorisation interpretation, and against the embodiment hypothesis. “*

Supplementary figure:

*“****Supplementary Figure S5. Intraparietal Sulcus (IPS) analysis.*** *(A) Univariate activations in prosthesis users. Results of the group level univariate contrast of (Active Prosthesis + Cosmetic Prosthesis) > Objects show that at the group level the IPS is also activated. (B) The IPS region of interest was taken from the Juelich Histological Atlas (30% probability of hIP1, hIP2, and hIP3). (C) Hand (left) and tool (right) distances from users’ ‘own’ prosthesis in IPS. Individual distances were normalised by the controls’ group mean distance, depending on the visual features of the ‘own’ prosthesis (hand-likeness). A value of one indicates similar hand/tool distance to controls. Users showed significantly greater distances between their own prosthesis and hands (t(25)=10.11, p<0.001contrary to the embodiment hypothesis. A significant increase in the distance of the ‘own’ prosthesis from tools was also observed (t(25)=4.62, p<0.001).”*

Lastly, we have also attempted an analysis in primary sensorimotor cortex. The rationale behind this analysis was the tight relationship previously observed between the sensorimotor and visuomotor hand regions (Tal et al., 2016; Van Den Heiligenberg et al., 2018). Univariate BOLD activity was negative and significantly different from zero for all 5 conditions, this encouraged us to run a multivariate analysis (since significant multivariate distances can be produced from negative activity (Diedrichsen, Wiestler, & Krakauer, 2013). However, as demonstrated in the table below, pairwise distances were not significantly different from zero, indicating that no reliable information could be extracted from the sensorimotor cortex using the present paradigm.

| **Univariate activity (z-statistic) in sensorimotor cortex for each condition** | | | | | |
| --- | --- | --- | --- | --- | --- |
|  | Hands | Tools | Active Prosthesis | Cosmetic Prosthesis | Own Prosthesis |
| Controls | -1.09 | -1.14 | -1.2 | -1.02 | - |
| T-test mean $\neq$ 0 | p<0.001 | p<0.001 | p<0.001 | p<0.001 | - |
| Prosthesis Users | -1.06 | -1.37 | -1.2 | -1.2 | -1.3 |
| T-test mean $\neq$ 0 | p<0.001 | p<0.001 | p<0.001 | p<0.001 | p<0.001 |

*values are average of the z-statistic across the ROI

| **Multivariate distances (RSA) in sensorimotor cortex** | | | | | |
| --- | --- | --- | --- | --- | --- |
|  | Hands ↔ Cosmetic Prosthesis | Hands ↔ Active Prosthesis | Tools ↔ Cosmetic Prosthesis | Tools ↔ Active Prosthesis | Cosmetic Prosthesis ↔ Active Prosthesis |
| Controls | -0.03 | 0.03 | 0.05 | 0.02 | 0.03 |
| T-test mean $\neq$ 0 | p=0.4 | p=0.32 | p=0.25 | p=0.57 | p=0.48 |
| Prosthesis Users | 0.04 | 0.04 | -0.02 | 0.01 | 0.07 |
| T-test mean $\neq$ 0 | p=0.26 | p=0.17 | p=0.63 | p=0.87 | p=0.04 |

*values are cross-nobis (LDC) distances; ↔ indicates pairwise distances.

3.8 The study included additional conditions like dismorphed images, lower limb and the own shoes (for people that were shown picture of their own prosthesis). What was the rational for not analysing these conditions which could provide important additional insights?

We now elaborate on the considerations leading us to discard the various conditions from the final analysis.

Methods (Stimuli section):

“*Additional conditions which were also included in the fMRI paradigm and initial analysis but not reported here were: dismorphed images (originally intended to account for low-level visual activity but discarded after univariate analysis revealed increased activity in OTC), and lower limbs (intended as a control body part but not included in final analysis).*

*“Shoes were selected as a familiar external object that was intended to exert similar cognitive effects (e.g. in terms of arousal) as the prosthesis, and therefore minimize differences in the scan time course across groups. Since we had no a priori interest in studying shoe representation, the shoe condition was not included in further analysis.”*

3.9 Distance measurement between brain patterns were computed using Cross validated Mahalanobis distances. Is it what is referred to as linear discriminant contrast (Walter 2016)?

Yes. This is specified both in the results and the methods sections. We have now specified LDC as well for clarity.

Methods (Representational similarity analysis):

*“Cross validated (leave-run-out) Mahalanobis distances (also known as LDC - linear discriminant contrast) [45,97] were then calculated between each pair of conditions.”*

3.10 The main hypotheses were tested by a series of group comparisons using t-tests. How were they corrected for multiple comparisons?

The table below provides a summary of our findings based on the nature of the test (planned; confirmatory; exploratory). Correction for multiple comparisons should be applied when exercising exploratory analysis, and when the planned comparisons are abundant. Confirmatory analyses do not require correction for multiple comparisons. We focused the manuscript on 4 group comparisons (embodiment, categorisation and the two ‘own’ indices) and 2 correlations with prosthesis usage. These were based on a priori predictions, were related to a few alternative planned-comparisons, and therefore did not require correction for multiple comparisons. We dissected some of these results, e.g. by considering potential confounds that might be driving the observed results (confirmatory analysis). Our exploratory analysis (relating to the role of prosthesis type and now also parietal representation) were corrected to multiple comparisons, unless if producing non-significant results.

This is now mentioned in the revised manuscript as follows:

Methods (Statistical analysis)

*“Corrections for multiple comparisons were included for exploratory analysis when resulting in significant differences, as indicated in the results section.”*

The table below lists all statistical tests in the paper and their type:

|  | Comparison | Result | Type | No. of related comparisons |
| --- | --- | --- | --- | --- |
| 1 | Hand-similarity group differences | t(54) = 0.47, p=0.64 | Planned | 2 (alternative – item 4) |
| 2 | Hand-similarity group differences for each hemisphere | Right OTC: t(54)=0.46 p=0.65, Left OTC: t(54)=0.54, p=0.60 | Confirmatory | Confirming 1 |
| 3 | Correlation of hand-similarity with usage | Pearson’s r(30)=-0.03, p=0.86 | Planned | 2 (alternative – item 6) |
| 4 | Categorisation group differences | t(54)=-2.55, p=0.01 | Planned | 2 (alternative - Item 1) |
| 5 | Prosthesis similarity group differences for each hemisphere | Right OTC: t(54)=-2.25 p=0.03, Left OTC: t(54)=-2.66, p=0.01 | Confirmatory | Confirming 4 |
| 6 | Prosthesis similarity correlation with usage | Pearson’s r(30)=0.43, p=0.01) | Planned | 2 (alternative - Item 3) |
| 7 | Prosthesis similarity cause of limb-loss ANCOVA | Group (F(1,29)=2.02, p=0.17) | Exploratory | Confirming 6 |
|  |  | usage effect (F(1,29)=4.11, p=0.052) | Confirmatory |  |
| 8 | Comparing the correlation of the two indices | z=-2.52, p=0.01 | Confirmatory | Confirming 6 |
| 9 | Prosthesis similarity comparison between prosthesis types | (t(20)=.055, p=.96) | Exploratory |  |
| 10 | Prosthesis similarity ANCOVA prosthesis types and Usage | prosthesis type effect (F(1,19)=.432, p=0.52) | Exploratory | Confirming 6 |
|  |  | usage effect (F(1,19)=6.01, p=0.02) | Confirmatory |  |
| 11 | Prosthesis similarity group comparison taking into account myo as active | (t(54)= -1.87, p=0.067) | Confirmatory | Confirming 4 |
| 12 | Prosthesis similarity correlation taking into account myo as active | (r(30)=0.48, p=0.006) | Confirmatory | Confirming 6 |
|  |  | (r(25)=0.46, p=0.015) (without them) |  |  |
| 13 | Own – hand distance | t(25)=2.85, p=0.009 | Planned |  |
| 14 | Own – Tool distance | t(25)=2.91, p=0.008 | Planned |  |
| 15 | Own – hand distance accounting for hand-like prostheses | t(25)=4.33, p=0.0002 | Confirmatory | Confirming 13 |
| 16 | Own – tool distance accounting for hand-like prostheses | t(25)=1.62, p=0.118 | Confirmatory | Confirming 14 |
| 17 | Parietal - hand-similarity index | t=0.71, p=0.48 | Exploratory |  |
| 18 | Parietal - prosthesis-similarity | t=-0.45, p=0.66 | Exploratory |  |
| 19 | Parietal - Own - hand distance | t=9.77, p<0.001 | Exploratory | Corrected for Multiple comparisons (2 - alternative item 20) |
| 20 | Parietal - Own - tool distance | t=5.32, p<0.001 | Exploratory | Corrected for Multiple comparisons (2 - alternative item 19) |

Reviewer #4: The manuscript is well written and describes an interesting set of data that challenges a widely-accepted viewpoint in the field, which is that upper limb prostheses are embodied as natural hands. The manuscript offers a new view, which is that prostheses are neurally represented as distinct from both hands and other tools. The manuscript also reports on prosthesis usage data and correlates prosthesis usage to neural representations. The manuscript discusses an important and timely topic, and has implications both for rehabilitation and human-machine interfacing.

Major Points:

4.1. My primary concern with the study is the underlying assumption that neural activity in visual areas of the brain is an appropriate indicator of embodiment. The study attempts to extend findings about neural responses in the occipitotemporal cortex (OTC) as evidence that prostheses are not embodied as a hand. However, to my knowledge, embodiment is generally discussed in the literature in the context of the body schema, which is a preconscious sensorimotor representation of the body. I would expect then, that neural responses in the sensory and motor cortices would be more appropriate to measure embodiment-related neural representations of prostheses, tools, and hands. Why did the authors choose to focus on OTC rather than any sensorimotor brain regions? I am not convinced that neural responses in visual brain areas are sufficient indicators of embodiment, and thus am not convinced that the conclusions are supported by the data. Please comment.

We fully agree with the referee that the lack of visual embodiment does not entail that the prosthesis cannot be embodied at another level, and especially at the level of the body schema. However, it is important to keep in mind that there are many types of embodiment, sensorimotor embodiment being only one among others. We have now clarified throughout the manuscript that we are probing visual embodiment, and not sensorimotor embodiment and apologise this wasn’t clear enough in the original version. An interesting case in this context is the rubber hand illusion (RHI), which is often taken as the paradigmatic example of embodiment. And yet some have argued that the rubber hand is not embodied at the sensorimotor level, but rather at the visual level (de Vignemont, 2018; Kammers et al., 2009). Furthermore, Tsakiris (2010) have argued that the key difference between the rubber hand and a tool and the reason for which one cannot have ownership for a tool is because of the lack of visual embodiment. Visual embodiment is thus conceived by some as a gateway for the sense of bodily ownership. And since the main challenge for amputees is to appropriate the prosthesis, we believe that visual embodiment is crucial. We have now reframed our introduction and discussion to highlight this perspective.

Introduction (2^nd^ paragraph):

*“Embodiment is an umbrella term for the multisensory association of external objects with body representation, which engages multiple levels from both conceptual and perceptual perspectives (see Discussion). From a neural standpoint, embodiment is defined by the successful allocation of brain resources, originally devoted to controlling one’s own body, to represent and operate external objects [17]. Visual embodiment is particularly relevant for studying prosthesis representation. This is because prosthesis usage is highly visually guided [18], and because visual embodiment has been suggested as a gateway for the sense of bodily ownership [19], a desired feature of prosthesis usage [9,10]. Previous efforts to test visual prosthesis embodiment have been centred on an illusion of body ownership over the prosthesis (most commonly, the rubber hand illusion, which relies on visual manipulations), with mixed success [11,20–23]. Crucially, such efforts focused on measuring visual embodiment but did not associate it with improved prosthesis usage [11,20–26] (though see [27] for results from two individuals). We have recently found that as a whole, one-handed individuals tend to respond neutrally to embodiment statements in relation to their own prostheses [28].”*

As now elaborated in our introduction, we have focused on the OTC, and EBA in particular, for multiple reasons:

Embodiment is a multisensory experience, which is predominantly measured by means of visual manipulations. For example, in the rubber hand illusion, vision is used to present both the rubber hand and the brush that is ‘capturing’ the felt touch. Of particular importance here, visual embodiment is conceived by some as a gateway for the sense of bodily ownership (Tsakiris, 2010). Moreover, prosthesis usage is highly visually-guided (Antfolk et al., 2013). As such, research of prosthesis embodiment should build on the importance of visual experience in daily prosthesis usage. An additional, more minor, consideration for us was that sensorimotor embodiment would be difficult to test since in congenital one-handers, the sensorimotor homunculus is architecturally changed compared with controls (Hahamy et al., 2017; Hahamy & Makin, 2019; Wesselink et al., 2019). Therefore, differences observed in these areas would be difficult to interpret.

Introduction (2^nd^ paragraph):

*“Embodiment is an umbrella term for the multisensory association of external objects with body representation, which engages multiple levels from both conceptual and perceptual perspectives (see Discussion). From a neural standpoint, embodiment is defined by the successful allocation of brain resources, originally devoted to controlling one’s own body, to represent and operate external objects [17]. Visual embodiment is particularly relevant for studying prosthesis representation. This is because prosthesis usage is highly visually guided [18], and because visual embodiment has been suggested as a gateway for the sense of bodily ownership [19], a desired feature of prosthesis usage [9,10]. Previous efforts to test visual prosthesis embodiment have been centred on an illusion of body ownership over the prosthesis (most commonly, the rubber hand illusion, which relies on visual manipulations), with mixed success [11,20–23].”*

EBA is a body- and hand-selective region, and is therefore suitable to testing embodiment (i.e. hand-like representation of artificial limbs). We (Orlov, Makin, & Zohary, 2010) and others (Bracci, Caramazza, & Peelen, 2015) have shown that this area selectively responds to body parts, including hands. EBA is not strictly visual, as it also contains sensorimotor attributes. We and others have shown that the region responds to hand movements (Astafiev, Stanley, Shulman, & Corbetta, 2004; Orlov, Porat, Makin, & Zohary, 2014) and passive touch (Tal, Geva, & Amedi, 2016). This is also very clearly demonstrated in blinds (Wesselink, Kikkert, Bridge, & Makin, 2018). EBA’s connectome further associates it with hand actions: it is uniquely connected to the sensorimotor hand area (Tal et al., 2016). We have recently shown that inter-modal connectivity between visual and motor hand-selective regions is modulated by the degree to which individuals use their prosthetic limb (Van Den Heiligenberg et al., 2018). This evidence indicates that this region is sensitive to daily prosthesis usage. Finally, and of particular relevance to our key research question, there is a known overlap between the hand-selective and tool-selective regions within the extrastriate area, making it an ideal brain area to test for the hypothesis that a prosthesis is represented like a tool (Bracci, Cavina-Pratesi, Ietswaart, Caramazza, & Peelen, 2012).

Introduction (3^rd^ paragraph):

*“The OTC is known to contain distinct visual representations of different object categories [30], for example: hands [31,32], tools [33], faces [34], and bodies [35]. It was previously shown to contain overlapping visual and motor body-part selective representations [31,36], and even responds to touch [37,38]. OTC’s connectome associates it with hand actions. For example, hand and body-selective visual areas uniquely connect to the primary sensorimotor hand area [37]. These characteristics qualify the OTC as an ideal candidate for studying action-related visual body-representation. We previously found that individuals who used their prosthesis more in daily lives also showed greater activity in OTC’s hand-selective visual areas when viewing images of prostheses, and greater functional coupling with sensorimotor hand areas [29]. This result demonstrates that prosthesis users are able to engage visuomotor hand-selective areas while (passively) representing a prosthesis.“*

Introduction (3^rd^ paragraph):

*“Alternatively, since the visual hand-selective regions partially overlap with hand-held tool representation [39], the observed activity gains may reflect the prosthesis being represented as a tool.“*

Furthermore, following the reviewers comments we also explored representation in parietal and motor areas, as well as additional ROIs in OTC. In short, we explored an additional ROI in OTC (based on visual hand representation), which reflected the same results as found in EBA (see supplementary results and Figure S4). We also performed analysis in the intraparietal sulcus where we found increased distances (i.e. “anti-embodiment” for users’ own prosthesis, relative to controls (as elaborated in the revised result section and Figure S5). Finally, we also explored activity patterns in M1, though unfortunately these were not interpretable (see our response to comment 1.3). We hope that this additional analysis helps better contextualise our findings for visual prosthesis representation.

Lastly, in the revised discussion we elaborate on the limitations of our approach, studying visual embodiment:

Discussion (1^st^ paragraph):

“*Despite benefiting from hand-selective cortical resources [29], the prosthesis is not ‘embodied’ into people’s visual hand representation. However, since the experimental design and analysis we implemented were a priori focused on visual hand embodiment in OTC, it is possible that other brain areas might find a different representational ‘solution’ to support the daily use of an artificial limb.”*

Discussion (4^th^ paragraph):

*“… Still, it is possible that other brain areas involved more directly in motor planning would produce other representational structures with respect to hand representation. Future research aimed at this question should take into consideration that at present, commercially available prosthesis motor control is fundamentally different from that of motor control of the hand, producing further potential challenges for sensorimotor embodiment”*

4.2.      In addition, given that sensorimotor representations of the body involve active control of a limb or tool and dynamic sensory feedback from the limb or tool, why was the experimental task designed to study passive visual experience rather than control or somatosensory feedback of the presented objects? I am not convinced that passively viewing an image of a prosthesis is a sufficient indication of the neural representation of the prosthesis during actual prosthesis usage. Please comment.

As highlighted above, prosthesis usage is massively visually guided, and together with the role of visual embodiment in agency, we wished to probe visual representations of the prostheses. We absolutely agree that we should aim to design our paradigms to capture behaviour more ecologically, and that prostheses usage is by definition non-passive. But please consider the various issues of studying prosthesis control in the MRI scanner. Beyond the principle technical issues (no metal is allowed inside the scanner; participants are lying on their backs; movements create multiple artefacts), we also encountered conceptual issues – people massively vary in their ability to use their prosthesis – making it very difficult to compare representation of usage (independent of other confounding factors, e.g. skill level, familiarity) across individuals. Similarly - how do we compare between the amputees and the controls? For these reasons, we were keen to use unfamiliar prosthetics as part of our design. Crucially, our resulting brain measures correlated significantly with everyday usage. This means that our measure, even if using non-ecologic settings, captures elementary aspects of prosthesis representation that are related to everyday actions. In other words, we agree that our task is not ecological but our task outcomes are ecologically relevant. Nevertheless, this key point is now highlighted in our discussion.

Discussion (4^th^ paragraph):

*“A second important consideration is that embodiment is a multisensory process, involving multiple brain areas beyond visual cortex [52]. Here we focused on visuomotor processing, and our experimental approach does have some limitations that should be considered. For example, our use of static images was designed to drive activity in the OTC, but not in other sensorimotor-related regions in the parietal and frontal regions [53], thereby limiting our conclusions to high-level visual cortex. The use of generic hand images that are not the participants’ own hands can also be limiting when approaching questions of embodiment. Here it is important to mention that despite using a profoundly non-ecological task in the scanner, the resulting brain representations, as captured with our prosthesis-similarity index, correlated significantly with the extent of everyday prosthesis usage. Therefore, despite the inherent limitations of our fMRI task, our task outcomes are ecologically relevant. Still, it is possible that other brain areas involved more directly in motor planning would produce other representational structures with respect to hand representation. Future research aimed at this question should take into consideration that at present, commercially available prosthesis motor control is fundamentally different from that of motor control of the hand, producing further potential challenges for sensorimotor embodiment.“*

4.3.      I believe that the perspective of the body-related images may have impacted the definition of the ROI and the measured neural responses to the hands and tools. One does not typically view one’s own body from a third-person perspective, but the study included both first- and third-person perspective images. What was the rationale for including third-person images when the goal was to assess embodiment, which relates to the sense of self? For the images shown in the third-person, I am concerned that the neural responses recorded might be related to viewing other people’s bodies rather than one’s own body.  Were there any differences in neural representations between images in the first- and third-person perspective? This seems to me to be a critical point. Furthermore, in Figure 1, I cannot distinguish which of the example images are in the first-person perspective because they are all floating in a solid background disconnected from bodies. Are the authors certain that the
images were interpreted by the participants as being in the first person? Please comment.

First, we wish to reassure the reviewer that our results did not strongly depend on our ROI definition (see our response to comment 4.1 above for further evidence from OTC and parietal cortex). With regards to our stimuli display, we apologise this important consideration wasn’t explained in the original manuscript. A key to our approach was the ability to compare representations across prosthesis users (congenital and acquired one-handers) and between prosthesis users and controls. As each participant was presented with their missing hand/prosthesis, it was crucial for our design to use multiple perspectives of both hands and prostheses. All images are available in open science framework (osf.io/kd2yh). Upon reflection we realise our description of first- and third-person perspectives was somewhat misleading, and in the revised manuscript we revised this to ‘multiple orientations’:

Methods(Stimuli section, 2^nd^ paragraph):

*“… each of the three prosthesis conditions involved multiple shots of a single prosthesis of different orientations. We chose to use multiple configurations of hands and prostheses to probe the experience of congenital one-handers and control participants (who mostly see prostheses/hands-of-the-missing-side from a 3rd person perspective, respectively). “*

Note that this design aspect is highly consistent with our own previous cognitive research showing adaptable categorisation of hands and tools in prosthesis users (van den Heiligenberg et al., 2017), as well as other’s research on visual hand representation in amelia patients (Striem-Amit et al., 2017). As the reviewer describes, these images are cropped from their native background and presented over a neutral background, as commonly practiced when studying OTC (for example: Downing, Jiang, Shuman, & Kanwisher, 2001; Kriegeskorte et al., 2008).

To our knowledge, currently there is no clear evidence that OTC/EBA represents allocentric and egocentric perspectives differently. There is previous evidence to suggest that the right EBA is more strongly activated by allocentric versus egocentric images (Saxe et al., 2006), or for self vs other body-part images (Myers & Sowden, 2008). But these reported effects were modest, and not well replicated (Hodzic et al., 2009). Regardless, since in our study we identify similar effects across hemispheres, we don’t believe we are impacted by these potential differences. We now convey this additional information in the revised manuscript.

Methods (Stimuli section, 2^nd^ paragraph):

*“We chose to use multiple configurations of hands and prostheses to probe the experience of congenital one-handers and control participants (who mostly see prostheses/hands-of-the-missing-side from a 3rd person perspective, respectively). We note that previous studies probing visual hand representation in similar populations [82], including specifically in OTC [54] used a similar approach. We further note that the few studies finding differences between egocentric/allocentric [84] or self/others [85] visual hand representation in OTC identified lateralised effects to the right OTC, whereas our effects are comparable across hemispheres.”*

Discussion (4^th^ paragraph):

*“The use of generic hand images that are not the participants’ own hands can also be limiting when approaching questions of embodiment. Here it is important to mention that despite using a profoundly non-ecological task in the scanner, the resulting brain representations, as captured with our prosthesis-similarity index, correlated significantly with the extent of everyday prosthesis usage. Therefore, despite the inherent limitations of our fMRI task, our task outcomes are ecologically relevant. Still, it is possible that other brain areas involved more directly in motor planning would produce other representational structures with respect to hand representation.”*

4.4.      The survey results regarding prosthesis usage are interesting and valuable. However, I believe that grouping these two metrics, frequency of wear and PAL, may not be appropriate. Users of cosmetic prostheses, for example, may wear their prostheses very frequently, but may not use them to perform any tasks. Although the authors note that the PAL has been validated, they do not present any indication that this combined prosthesis usage score has been validated. Could the authors please provide information about the validation of this combined usage metric?

We apologise for glossing over this hugely important point, which we elaborated on in our previous manuscript, and reiterate in the revised manuscript. In our dataset, frequent wear-time was strongly correlated with better utility in daily tasks (PAL) (r(30)=.84; p<.001). This is because, contrary to its reputation, the active prostheses (the ones providing actuation of grip) do not necessarily afford greater functionality, in terms of daily life function, than the cosmetic ones. This is reflected in the fact that one-handers tend to prefer the cosmetic prostheses for daily wear (Jang et al., 2011), and further report greater satisfaction with the functionality of cosmetic prostheses (Østlie et al., 2012). This is nicely exemplified in the current study cohort — we compared PAL scores between cosmetic and active prosthesis users (participants were assigned to each group based on the prosthesis type they use more in daily life). This analysis shows that users tend to incorporate a cosmetic prosthesis more in daily tasks (though not significantly so), as reflected in the PAL scores: average active PAL ± s.e.m. = 0.19±0.06; average cosmetic PAL = 0.25+0.06; group difference p=0.50).

I would also be interested to see the analysis comparing hand-similarity index to prosthesis usage broken down for the two components of the usage metric: i.e. one plot of hand-similarity index to wear frequency and one plot of hand-similarity index to PAL score. Did the authors attempt to decouple the two usage metrics?

We haven’t so far, because we believe successful prosthesis usage is a combination of both wear time and utility, and because the correlation between these two measures is so high. Following this comment, we did run the analysis separately and found no significant for each of the metrics with the hand-similarity index (Wear frequency: r(30)=0.083, p=0.65; PAL: r(30)= 0.05, p=0.78). However, both metrics did show significant correlation with the prosthesis-similarity index (Wear frequency: r(30)=0.46, p=0.01; PAL: r(30)=0.51, p=0.003). We also attach a plot of the two significant correlations:

4.5.  I found the discussion about visual expertise to be very interesting in relation to the conclusions of this study. It seems to me that the emergence of prosthesis-specific visual expertise in prosthesis users is well-supported by the results, and perhaps a better interpretation of the data than a lack of embodiment. The authors discuss prior literature on car experts, who show increased activity in object-selective areas of OTC compared to non-experts. In this context, the prosthesis users could be considered prosthesis experts, since they have more experience with prostheses than control participants (perhaps except in rare cases). If car-specific regions of the OTC emerge through visual learning, it is reasonable that prosthesis-specific regions could also emerge through visual learning. If there were a ‘prosthesis-specific area’ of the OTC, then it could follow that prosthesis experts would have increased activation of this area than non-experts. This prosthesis-specific region of the OTC could be considered to be the non-hand, non-tool region the authors observed in their fMRI data. It would also make sense that prosthesis usage would correlate with the emergence of the prosthesis-specific region in the OTC, since visual learning would be promoted by increased visual exposure to prostheses (as the prosthesis is used more over time). However, I do not think that this visual learning interpretation implies that prostheses are not embodied, because visual learning could occur independently from sensorimotor learning, and to my understanding, sensorimotor learning is intricately related to embodiment.

We agree with the reviewer’s interpretation relating to the role of visuomotor expertise, while using a prosthesis in daily life, in forming prosthesis categorisation in the OTC (with the clarification that we are not measuring net increases in activity but rather a change of the representational structure). But we wish to note that the categorisation model should not be viewed as an opposite, but rather an alternative model to the embodiment model. We explicitly tested for embodiment in the most straightforward way we could think of – does your own prosthesis evoke a representation that is similar to that of a hand? The clear answer is – NO. We found evidence to support the opposite – users’ own prosthesis representation becomes more dissimilar to that of a hand. This finding is now further consolidated by similar results in parietal cortex and similar results across ROIs in the OTC (see response to comment 4.1). Why this is the case is open for interpretation, which we now elaborate on in the discussion. And we agree that this doesn’t exclude the possibility that other forms of embodiment (e.g. in motor cortex) could still be achieved (this point is also now included in the revised manuscript). However, as stated above, this is incredibly challenging to test for practical reasons. It would also be difficult to interpret, first and foremost because hand representation in the motor cortex is persistent in amputees and non-existent in congenital one-handers. Therefore, the visual analysis we carry our here is the most comprehensive examination to date for implicit visual embodiment. In the revised manuscript we have completely reformulated our discussion to address these key interpretations more comprehensively. Throughout the manuscript we also now emphasize that we are testing for **visual** embodiment.

With regards to the differences between visual and sensorimotor learning, unfortunately, one cannot acquire motor experience with using a prosthetic arm without concurrent visual experience. In fact, as we highlight in the manuscript, prosthesis usage is notoriously over-reliant on visual feedback. Therefore, for our model (prosthesis usage), visual and motor expertise are inherently linked and as such we did not provide additional controls to dissociate them.

Discussion (1^st^-5^th^ paragraphs):

*“Together with preliminary results showing that prosthesis users exhibited a less hand-like representation of their own prosthesis in parietal cortex, our results collectively show that neural visual embodiment does not predict successful adoption of wearable technology by the human brain. Despite benefiting from hand-selective cortical resources [29], the prosthesis is not ‘embodied’ into people’s visual hand representation. However, since the experimental design and analysis we implemented were a priori focused on visual hand embodiment in OTC, it is possible that other brain areas might find a different representational ‘solution’ to support the daily use of an artificial limb.*

*In the current study we did not find evidence for neural visual embodiment (hand-like prosthesis representation) in prosthesis-users or a relationship between visual embodiment and prosthesis usage. As stated above, an intuitive and increasingly popular view that has been inspiring biomedical design and innovation is that embodiment will optimise the usage of technology, such as an upper-limb prostheses [10,11,13–16,50,51]. How can these two views be resolved?*

*One potential solution originates from the fact that embodiment is a multifaced phenomenon [17], with distinct levels (i.e. phenomenological – does the prosthesis feel like my hand? cognitive – do I react to/with the prosthesis like I would to/with my own hand?; neural – is the prosthesis represented via the same mechanisms as a hand? [7]). In another recent study where we probed the phenomenological and cognitive levels of prosthesis embodiment, we found it to correlate with prosthesis usage [28]. Here we focused our research on the neural level, since it is entirely unknown whether objective representation of the prosthesis as a body-part associates with prosthesis acceptance and usage, let alone benefits it. It is possible, and even likely, that embodiment manifests differently in each of these distinct levels, which may also vary in their importance or even relevance for successful technological implementation [7].*

*A second important consideration is that embodiment is a multisensory process, involving multiple brain areas beyond visual cortex [52]. Here we focused on visuomotor processing, and our experimental approach does have some limitations that should be considered. For example, our use of static images was designed to drive activity in the OTC, but not in other sensorimotor-related regions in the parietal and frontal regions [53], thereby limiting our conclusions to high-level visual cortex. The use of generic hand images that are not the participants’ own hands can also be limiting when approaching questions of embodiment. Here it is important to mention that despite using a profoundly non-ecological task in the scanner, the resulting brain representations, as captured with our prosthesis-similarity index, correlated significantly with the extent of everyday prosthesis usage. Therefore, despite the inherent limitations of our fMRI task, our task outcomes are ecologically relevant. Still, it is possible that other brain areas involved more directly in motor planning would produce other representational structures with respect to hand representation. Future research aimed at this question should take into consideration that at present, commercially available prosthesis motor control is fundamentally different from that of motor control of the hand, producing further potential challenges for sensorimotor embodiment.*

*Thirdly, and most speculatively, it is possible that the prosthesis may still be represented in OTC as a body part, but one that is not a hand. After all, prosthesis users have strong semantic and sensorimotor knowledge of a hand (all users in the study had one intact hand; acquired amputees had prior experience of their missing hand, including lingering phantom sensations; see also [54] for related findings showing normal visual hand representation in individuals born without both hands). Their experience with operation of a prosthesis is fundamentally different from that of a hand. If body representation is not strictly tuned to the specific fine-grained features of a given body part (e.g. the digits of the hand), but is instead also represented at a higher level (e.g. effectors [55] or based on other functionality features [56]), then the dissociation of prostheses from hand representation observed in our study should not be taken as evidence for lack of embodiment per se, but rather lack of embodiment as a hand. Therefore, we propose that future studies of artificial limb embodiment should not be limited to identifying and/or quantifying hand representation (as the current common practice, e.g. using the rubber hand illusion).*

Finally, we wish to point out that considering the prominence of the embodiment theory across the multiple disciplines engaged with prosthesis design, usage and representation, we feel strongly that even if not offering a sweeping answer to how the entire brain represents artificial limbs, our findings provide an important alternative that needs to be considered. This sentiment is now highlighted in our concluding remarks, as follows:

Discussion (9^th^ paragraph):

*“To conclude, we provide a novel neural correlate for the adoption of wearable technology that is distinct from visual embodiment of a prosthesis as a hand. Successful prosthesis usage, in terms of both wear-time and habit in daily life, was predicted not by visual embodiment (hand-similarity), but by a more distinct categorical representation of artificial limbs. Understanding whether the brain can treat a prosthesis as a hand, and whether this hand-like representation provides a real advantage for prosthesis users, will have important implications on future design and assessment of wearable technology. Considering the limitations related to our focus on visual prosthesis representation in passive settings we are currently unable to offer a sweeping answer to how the entire brain represents artificial limbs. However, our findings provide an important alternative to the highly prominent embodiment theory that needs to be considered. As such, much more research is necessary to provide a comprehensive understanding of the neural basis of successful prosthesis usage in the human brain.“*

Minor Points:

4.6.      The manuscript needs to be revised throughout for typos.

Our sincere apologies – we have previously requested a native English speaker to proof-read the manuscript prior to submission, but apparently, they were not comprehensive in their work. We have now asked an additional individual to read through the manuscript prior to resubmission.

4.7.      I am concerned about the way in which the ROI was defined based on the presentation of headless-bodies versus everyday objects. Why were the bodies headless? Could the unnaturalness or dehumanizing nature of the images of headless-bodies impact arousal or emotion-related centers of the brain?

As elaborated in our response to comment 4.1, our results are not confounded by the ROI selection criteria. As to why we used headless bodies - this is a gold standard for how to localise EBA (Downing & Peelen, 2016; Pitcher, Charles, Devlin, Walsh, & Duchaine, 2009), and to assure the readers/experts that we are indeed localising EBA we had to stick with this standard. Note that we did not want to use the individual subject’s hand ROI due to issues of circular analysis (Kriegeskorte, Simmons, Bellgowan, & Baker, 2009). In the revised manuscript we have also added analyses from two additional ROIs within the OTC showing similar results, as elaborated above.

4.8.      For viewing images of prostheses, was the handedness of the prosthesis images matched to the handedness of the prosthesis user’s limb loss? For example, for a person with an amputation of the right arm, were the observed images of right-handed prostheses? Could the authors please comment on how handedness may have impacted the results?

Yes, we matched the side of the missing hand with that of the prosthesis, as we detail in the manuscript:

Methods (Stimuli):

*“For hands and prosthesis images, the effector was matched to the prosthesis-users’ missing-hand side or the non-dominant hand in controls (e.g. participants missing their left hand were presented with ‘left-handed’ hands/prostheses).”*

Following the reviewer’s comment, we have now repeated the prosthesis-similarity index group analysis, adding side of missing hand (or handedness in controls) as an additional factor, using a mixed-level ANOVA. We found no significant effect of side [F(1,52)=0.32, p=0.58] with a close-to-significant group effect [F(1,52)=3.87, p=0.055]. This result indicates that the laterality of the viewed stimuli did not impact our findings. We also conducted a t-test within the prosthesis-users group to make sure side of amputation specifically did not have a significant effect on the prosthesis-similarity index. We found no significant difference between individuals with left (n=24) and right (n=8) limb loss [t(30)=0.9 p=0.37] (Though this should be interpreted with caution due to the low number of individuals missing a right hand).

4.9.      In the introduction, the authors suggest that prosthetic limbs are the “most established form of wearable technology to date.” While I agree that prosthetic limbs are an important category of wearable technology, many other wearable technologies, such as hearing aids, glasses, and watches, are much more widely adopted than prostheses.

We thank the reviewer for pointing this out to us, we have now revised this to “wearable motor technology”

4.10.      In the Discussion, the authors note that the categorization hypothesis is “well validated in neuroscience” but do not present any citations to demonstrate prior instances of this categorization hypothesis in the literature. Please provide citations for this prior work.

We now mention in this sentence that this will be detailed later. We have dedicated a full paragraph with many references to support this argument:

Introduction (3^rd^ paragraph):

*“As a third option, since object categorisation in OTC is thought to be based on their semantic and functional properties[40], expert prosthesis usage may result in the emergence of prostheses representation as a new ‘category’, diverging from its existing natural categories (e.g. ‘hands’, ‘tools’). This third alternative is consistent with recent evidence showing that visual expertise can contribute to the shaping of categorical representation in OTC [41] (see Discussion).”*

Discussion (6^th^ paragraph):

*“Instead of visual hand embodiment, we found that a significant amount of individual differences in prosthesis usage can be predicted by the extent of prosthesis categorisation within the visual body-selective area, providing a significantly better model than hand-embodiment. This result is also consistent with the known organising principle of the OTC, where categorical representation reflects our knowledge of grouping and patterns, which are not necessarily present in the bottom-up sensory inputs [40,57,58]. Moreover, categorical representation in OTC was shown to reflect individual differences in conceptual knowledge [59]. Accordingly, people who acquire a specific visual expertise, such as car experts, show increased activity in object-selective areas in OTC (for a review see [60]). Research on object-experts, therefore, provides compelling evidence for the role of visual learning and experience in shaping and fine-tuning categorical representation. Although various studies have demonstrated a relation between expertise and activation, few studies performed multivariate analyses, and those that did reported mixed results [61–64]. For example, Martens and colleagues (2018) found that expertise did not alter the representational configuration of the category of expertise, while McGugin and colleagues (2015) who studied car representation of experts in the Fusiform Face Area, report that car representation became more similar to that of faces with expertise. In this context, our present results provide a novel perspective on visual expertise. This is because our results show divergence of prosthesis representation from the ‘natural’ categories normally existing in this brain area, consistent with the formation of a new categorical representation. In other words, rather than refining a category, prosthesis usage results in the formation of a new category. Gomez and colleagues (2019) recently reported that childhood experience with playing a Pokémon videogame relates to increased classification of Pokémon, compared with other related categories, in the ventral occipital cortex [41]. Extending this finding, we report that categorical prosthesis representation correlates with (ongoing) visuomotor experience in adulthood. As these effects were found in both congenital and acquired one-handers, this prosthesis categorisation effect doesn’t seem to relate to a specific developmental period. “*

**References**

Antfolk, C., D’Alonzo, M., Rosén, B., Lundborg, G., Sebelius, F., & Cipriani, C. (2013). Sensory feedback in upper limb prosthetics. *Expert Review of Medical Devices*, *10*(1), 45–54. https://doi.org/10.1586/erd.12.68

Astafiev, S. V, Stanley, C. M., Shulman, G. L., & Corbetta, M. (2004). Extrastriate body area in human occipital cortex responds to the performance of motor actions. *Nature Neuroscience*, *7*(5), 542–548. https://doi.org/10.1038/nn1241

Beauchamp, M. S., LaConte, S., & Yasar, N. (2009). Distributed representation of single touches in somatosensory and visual cortex. *Human Brain Mapping*, *30*(10), 3163–3171. https://doi.org/10.1002/hbm.20735

Beckerle, P., Kirchner, E. A., Christ, O., Kim, S.-P., Shokur, S., Dumont, A. S., … Lenggenhager, B. (2018). Feel-Good Robotics: Requirements on Touch for Embodiment in Assistive Robotics. *Frontiers in Nerorobotics*, *12*(84), 1–7. https://doi.org/10.3389/fnbot.2018.00084

Bensmaia, S. J., & Miller, L. E. (2014). Restoring sensorimotor function through intracortical interfaces: progress and looming challenges. *Nature Reviews Neuroscience*, *15*(5), 313–325. https://doi.org/10.1038/nrn3724

Bilalić, M., Grottenthaler, T., Nägele, T., & Lindig, T. (2016). The Faces in Radiological Images: Fusiform Face Area Supports Radiological Expertise. *Cerebral Cortex*, *26*(3), 1004–1014. https://doi.org/10.1093/cercor/bhu272

Bracci, S., Caramazza, A., & Peelen, M. V. (2015). Representational Similarity of Body Parts in Human Occipitotemporal Cortex. *The Journal of Neuroscience*, *35*(37), 12977–12985. https://doi.org/10.1523/JNEUROSCI.4698-14.2015

Bracci, S., Cavina-Pratesi, C., Ietswaart, M., Caramazza, A., & Peelen, M. V. (2012). Closely overlapping responses to tools and hands in left lateral occipitotemporal cortex. *Journal of Neurophysiology*, *107*(5), 1443–1456. https://doi.org/10.1152/jn.00619.2011

Bracci, S., & Peelen, M. V. (2013). Body and object effectors: The organization of object representations in high-level visual cortex reflects body-object interactions. *Journal of Neuroscience*, *33*(46), 18247–18258. https://doi.org/10.1523/JNEUROSCI.1322-13.2013

Braunlich, K., Liu, Z., & Seger, C. A. (2017). Occipitotemporal Category Representations Are Sensitive to Abstract Category Boundaries Defined by Generalization Demands. *The Journal of Neuroscience*, *37*(32), 7631–7642. https://doi.org/10.1523/JNEUROSCI.3825-16.2017

Braunlich, K., & Love, B. C. (2018). Occipitotemporal representations reflect individual differences in conceptual knowledge. *Journal of Experimental Psychology: General*, *Advance on*. https://doi.org/10.1037/xge0000501

Collins, K. L., Guterstam, A., Cronin, J., Olson, J. D., Ehrsson, H. H., & Ojemann, J. G. (2016). Ownership of an artificial limb induced by electrical brain stimulation. *PNAS*. https://doi.org/10.1073/pnas.1616305114

D’Alonzo, M., Clemente, F., & Cipriani, C. (2015). Vibrotactile Stimulation Promotes Embodiment of an Alien Hand in Amputees With Phantom Sensations. *IEEE Transactions on Neural Systems and Rehabilitation Engineering*, *23*(3), 450–457. https://doi.org/10.1109/TNSRE.2014.2337952

de Oliveira Barata, S., Clode, D., Taylor, J., & Elias, H. (2017). Vine Arm. Retrieved from http://www.thealternativelimbproject.com/project/vine/

de Vignemont, F. (2018). *Mind the body: An exploration of bodily self-awareness*. *Mind the Body: An Exploration of Bodily Self-Awareness*. Oxford University Press. https://doi.org/10.1093/oso/9780198735885.001.0001

De Vignemont, F. (2011). Embodiment, ownership and disownership. *Consciousness and Cognition*, *20*(1), 82–93. https://doi.org/10.1016/j.concog.2010.09.004

Dempsey-Jones, H., Wesselink, D. B., Friedman, J., & Makin, T. R. (2019). Organized Toe Maps in Extreme Foot Users. *Cell Reports*, *28*(11), 2748-2756.e4. https://doi.org/10.1016/j.celrep.2019.08.027

Diedrichsen, J., Wiestler, T., & Krakauer, J. W. (2013). Two distinct ipsilateral cortical representations for individuated finger movements. *Cerebral Cortex*, *23*(6), 1362–1377. https://doi.org/10.1093/cercor/bhs120

Downing, P. E., Jiang, Y., Shuman, M., & Kanwisher, N. (2001). A cortical area selective for visual processing of the human body. *Science*, *293*(5539), 2470–2473. https://doi.org/10.1126/science.1063414

Downing, P. E., & Peelen, M. V. (2016). Body selectivity in occipitotemporal cortex: Causal evidence. *Neuropsychologia*, *83*, 138–148. https://doi.org/10.1016/j.neuropsychologia.2015.05.033

Ehrsson, H. H., Rosén, B., Stockselius, A., Ragnö, C., Köhler, P., & Lundborg, G. (2008). Upper limb amputees can be induced to experience a rubber hand as their own. *Brain : A Journal of Neurology*, *131*(Pt 12), 3443–3452. https://doi.org/10.1093/brain/awn297

Giummarra, M. J., Gibson, S. J., Georgiou-Karistianis, N., & Bradshaw, J. L. (2008, January 1). Mechanisms underlying embodiment, disembodiment and loss of embodiment. *Neuroscience and Biobehavioral Reviews*. Pergamon. https://doi.org/10.1016/j.neubiorev.2007.07.001

Gomez, J., Barnett, M., & Grill-Spector, K. (2019). Extensive childhood experience with Pokémon suggests eccentricity drives organization of visual cortex. *Nature Human Behaviour*, 1. https://doi.org/10.1038/s41562-019-0592-8

Graczyk, E. L., Resnik, L., Schiefer, M. A., Schmitt, M. S., & Tyler, D. J. (2018). Home use of a neural-connected sensory prosthesis provides the functional and psychosocial experience of having a hand again. *Scientific Reports*, *8*(1), 1–17. https://doi.org/10.1038/s41598-018-26952-x

Hahamy, A., Macdonald, S. N., van den Heiligenberg, F. M. Z., Kieliba, P., Emir, U., Malach, R., … Makin, T. R. (2017). Representation of Multiple Body Parts in the Missing-Hand Territory of Congenital One-Handers. *Current Biology*, *27*(9), 1350–1355. https://doi.org/10.1016/j.cub.2017.03.053

Hahamy, A., & Makin, T. R. (2019). Remapping in cerebral and cerebellar cortices is not restricted by somatotopy. *The Journal of Neuroscience*, 2599–18. https://doi.org/10.1523/jneurosci.2599-18.2019

Hellman, R. B., Chang, E., Tanner, J., Helms Tillery, S. I., & Santos, V. J. (2015). A Robot Hand Testbed Designed for Enhancing Embodiment and Functional Neurorehabilitation of Body Schema in Subjects with Upper Limb Impairment or Loss. *Frontiers in Human Neuroscience*, *9*, 26. https://doi.org/10.3389/fnhum.2015.00026

Hodzic, A., Muckli, L., Singer, W., & Stirn, A. (2009). Cortical responses to self and others. *Human Brain Mapping*, *30*(3), 951–962. https://doi.org/10.1002/hbm.20558

Jang, C. H., Yang, H. S., Yang, H. E., Lee, S. Y., Kwon, J. W., Yun, B. D., … Jeong, H. W. (2011). A Survey on Activities of Daily Living and Occupations of Upper Extremity Amputees. *Annals of Rehabilitation Medicine*, *35*, 907. https://doi.org/10.5535/arm.2011.35.6.907

Kammers, M. P. M., Longo, M. R., Tsakiris, M., Dijkerman, H. C., & Haggard, P. (2009). Specificity and Coherence of Body Representations. *Perception*, *38*(12), 1804–1820. https://doi.org/10.1068/p6389

Kriegeskorte, N., Mur, M., Ruff, D. A., Kiani, R., Bodurka, J., Esteky, H., … Bandettini, P. A. (2008). Matching Categorical Object Representations in Inferior Temporal Cortex of Man and Monkey. *Neuron*, *60*(6), 1126–1141. https://doi.org/10.1016/J.NEURON.2008.10.043

Kriegeskorte, N., Simmons, W. K., Bellgowan, P. S., & Baker, C. I. (2009). Circular analysis in systems neuroscience: The dangers of double dipping. *Nature Neuroscience*, *12*(5), 535–540. https://doi.org/10.1038/nn.2303

Longo, M. R., Sadibolova, R., & Tamè, L. (2016). Embodying prostheses – how to let the body welcome assistive devices: Comment on “The embodiment of assistive devices—from wheelchair to exoskeleton” by M. Pazzaglia and M. Molinari. *Physics of Life Reviews*, *16*, 184–185. https://doi.org/10.1016/J.PLREV.2016.01.012

Marasco, P. D., Hebert, J. S., Sensinger, J. W., Shell, C. E., Schofield, J. S., Thumser, Z. C., … Orzell, B. M. (2018). Illusory movement perception improves motor control for prosthetic hands. *Science Translational Medicine*, *10*(432), 1–13. https://doi.org/10.1126/scitranslmed.aao6990

Marasco, P. D., Kim, K., Colgate, J. E., Peshkin, M. A., & Kuiken, T. A. (2011). Robotic touch shifts perception of embodiment to a prosthesis in targeted reinnervation amputees. *Brain : A Journal of Neurology*, *134*(Pt 3), 747–758. https://doi.org/10.1093/brain/awq361

Martens, F., Bulthé, J., van Vliet, C., & Op de Beeck, H. (2018). Domain-general and domain-specific neural changes underlying visual expertise. *NeuroImage*, *169*, 80–93. https://doi.org/10.1016/J.NEUROIMAGE.2017.12.013

McGugin, R. W., Van Gulick, A. E., Tamber-Rosenau, B. J., Ross, D. A., & Gauthier, I. (2015). Expertise Effects in Face-Selective Areas are Robust to Clutter and Diverted Attention, but not to Competition. *Cerebral Cortex*, *25*, 26610–2622. https://doi.org/10.1093/cercor/bhu060

Mulvey, M. R., Fawkner, H. J., Radford, H. E., & Johnson, M. I. (2012). Perceptual Embodiment of Prosthetic Limbs by Transcutaneous Electrical Nerve Stimulation. *Neuromodulation: Technology at the Neural Interface*, *15*(1), 42–47. https://doi.org/10.1111/j.1525-1403.2011.00408.x

Myers, A., & Sowden, P. T. (2008). Your hand or mine? The extrastriate body area. *NeuroImage*, *42*(4), 1669–1677. https://doi.org/10.1016/j.neuroimage.2008.05.045

Nili, H., Wingfield, C., Walther, A., Su, L., Marslen-Wilson, W., & Kriegeskorte, N. (2014). A Toolbox for Representational Similarity Analysis. *PLoS Computational Biology*, *10*(4). https://doi.org/10.1371/journal.pcbi.1003553

Orlov, T., Makin, T. R., & Zohary, E. (2010). Topographic Representation of the Human Body in the Occipitotemporal Cortex. *Neuron*, *68*(3), 586–600. https://doi.org/10.1016/j.neuron.2010.09.032

Orlov, T., Porat, Y., Makin, T. R., & Zohary, E. (2014). Hands in motion: An upper-limb-selective area in the occipitotemporal cortex shows sensitivity to viewed hand kinematics. *Journal of Neuroscience*, *34*(14), 4882–4895. https://doi.org/10.1523/JNEUROSCI.3352-13.2014

Østlie, K., Lesjø, I. M., Franklin, R. J., Garfelt, B., Skjeldal, O. H., & Magnus, P. (2012). Prosthesis rejection in acquired major upper-limb amputees: a population-based survey. *Disability and Rehabilitation: Assistive Technology*, *7*(4), 294–303. https://doi.org/10.3109/17483107.2011.635405

Pazzaglia, M., & Molinari, M. (2016, March 1). The embodiment of assistive devices-from wheelchair to exoskeleton. *Physics of Life Reviews*. Elsevier. https://doi.org/10.1016/j.plrev.2015.11.006

Pitcher, D., Charles, L., Devlin, J. T., Walsh, V., & Duchaine, B. (2009). Triple Dissociation of Faces, Bodies, and Objects in Extrastriate Cortex. *Current Biology*, *19*(4), 319–324. https://doi.org/10.1016/j.cub.2009.01.007

Reddy, L., & Kanwisher, N. (2006). Coding of visual objects in the ventral stream. *Current Opinion in Neurobiology*, *16*(4), 408–414. https://doi.org/10.1016/J.CONB.2006.06.004

Rognini, G., Petrini, F. M., Raspopovic, S., Valle, G., Granata, G., Strauss, I., … Blanke, O. (2019, August 12). Multisensory bionic limb to achieve prosthesis embodiment and reduce distorted phantom limb perceptions. *Journal of Neurology, Neurosurgery and Psychiatry*. BMJ Publishing Group Ltd. https://doi.org/10.1136/jnnp-2018-318570

Rosén, B., Ehrsson, H. H., Antfolk, C., Cipriani, C., Sebelius, F., Lundborg, G., … Lundborg, G. (2009). Referral of sensation to an advanced humanoid robotic hand prosthesis Referral of sensation to an advanced humanoid robotic hand prosthesis. *Scandinavian Journal of Plastic and Reconstructive Surgery and Hand Surgery* , *43*, 260–266. https://doi.org/10.3109/02844310903113107

Ross, D. A., Tamber-Rosenau, B. J., Palmeri, T. J., Zhang, J., Xu, Y., & Gauthier, I. (2018). High-resolution Functional Magnetic Resonance Imaging Reveals Configural Processing of Cars in Right Anterior Fusiform Face Area of Car Experts. *Journal of Cognitive Neuroscience*, *30*(7), 973–984. https://doi.org/10.1162/jocn_a_01256

Saxe, R., Jamal, N., & Powell, L. (2006). My body or yours? The effect of visual perspective on cortical body representations. *Cerebral Cortex*, *16*(2), 178–182. https://doi.org/10.1093/cercor/bhi095

Schmalzl, L., Kalckert, A., Ragnö, C., & Ehrsson, H. H. (2014). Neural correlates of the rubber hand illusion in amputees: A report of two cases. *Neurocase*, *20*(4), 407–420. https://doi.org/10.1080/13554794.2013.791861

Seger, C. A., & Miller, E. K. (2010). Category Learning in the Brain. https://doi.org/10.1146/annurev.neuro.051508.135546

Striem-Amit, E., Vannuscorps, G., & Caramazza, A. (2017). Sensorimotor-independent development of hands and tools selectivity in the visual cortex. *Proceedings of the National Academy of Sciences of the United States of America*, *114*(18), 4787–4792. https://doi.org/10.1073/pnas.1620289114

Tal, Z., Geva, R., & Amedi, A. (2016). The origins of metamodality in visual object area LO: Bodily topographical biases and increased functional connectivity to S1. *NeuroImage*, *127*, 363–375. https://doi.org/10.1016/j.neuroimage.2015.11.058

Tsakiris, M. (2010). My body in the brain: A neurocognitive model of body-ownership. *Neuropsychologia*, *48*(3), 703–712. https://doi.org/10.1016/j.neuropsychologia.2009.09.034

Tsao, D. Y., & Livingstone, M. S. (2008). Mechanisms of Face Perception. *Annual Review of Neuroscience*, *31*(1), 411–437. https://doi.org/10.1146/annurev.neuro.30.051606.094238

Tyler, D. J. (2015). Neural interfaces for somatosensory feedback: bringing life to a prosthesis. *Current Opinion in Neurology*, *28*(6), 574–581. https://doi.org/10.1097/WCO.0000000000000266

Valle, G., Mazzoni, A., Iberite, F., D’Anna, E., Strauss, I., Granata, G., … Micera, S. (2018). Biomimetic Intraneural Sensory Feedback Enhances Sensation Naturalness, Tactile Sensitivity, and Manual Dexterity in a Bidirectional Prosthesis. *Neuron*, *100*(1), 37-45.e7. https://doi.org/10.1016/J.NEURON.2018.08.033

Van Den Heiligenberg, F. M. Z., Orlov, T., MacDonald, S. N., Duff, E. P., Henderson Slater, D., Beckmann, C. F., … Makin, T. R. (2018). Artificial limb representation in amputees. *Brain*, *141*(5), 1422–1433. https://doi.org/10.1093/brain/awy054

van den Heiligenberg, F. M. Z., Yeung, N., Brugger, P., Culham, J. C., & Makin, T. R. (2017). Adaptable Categorization of Hands and Tools in Prosthesis Users. *Psychological Science*, *28*, 395–398. https://doi.org/10.1177/0956797616685869

Walther, A., Nili, H., Ejaz, N., Alink, A., Kriegeskorte, N., & Diedrichsen, J. (2016). Reliability of dissimilarity measures for multi-voxel pattern analysis. *NeuroImage*, *137*, 188–200. https://doi.org/10.1016/J.NEUROIMAGE.2015.12.012

Wesselink, D. B., Kikkert, S., Bridge, H., & Makin, T. (2018). Sensorimotor finger-specific information in the cortex of the congenitally blind. *Journal of Vision*, *18*(10). https://doi.org/10.1167/18.10.99

Wesselink, D. B., van den Heiligenberg, F. M., Ejaz, N., Dempsey-Jones, H., Cardinali, L., Tarall-Jozwiak, A., … Makin, T. R. (2019). Obtaining and maintaining cortical hand representation as evidenced from acquired and congenital handlessness. *ELife*, *8*(e37227), 1–19. https://doi.org/10.7554/eLife.37227
