# Supplementary material for: Is an artificial limb embodied as a hand? Brain decoding in prosthetic limb users
Source: PLoS Biol. 2020 Jun 8;18(6):e3000729. doi: 10.1371/journal.pbio.3000729 (PMC7302856; doi:10.1371/journal.pbio.3000729)
Supplement: Attachment — Submitted filename: Rebuttal_rev2_submitted.docx [file pbio.3000729.s012.docx]

Dear Professor Gasque,

We are pleased to provide the requested revisions to our manuscript. We have addressed all of the issues raised by the reviewers, as elaborated in the point-by-point responses below (original comments in blue italics, our responses in black and text inserts in black italics). In particular, we included additional data-driven analysis on the individual pair-wise distances to provide further evidence on the interactions driving the observed categorisation effect. We feel confident that our revisions will fully satisfy the reviewers.

REVIEWS:

Reviewer #1: The authors have provided extensive replies and significantly expanded on the original manuscript in the first round of corrections. They have convincingly made the claim that their "categorization hypothesis" (that prostheses become their own semantic category) provides a better explanation than the traditional embodiment hypothesis (that protheses are represented more like hands). At the same time, the selected paradigm does support complete falsification of the embodiment hypothesis. fMRI activation patterns in the OTC during passive viewing of prostheses may not be the optimal measure to make claims about the embodied representation that would invariably involve widespread brain areas including the motor and somatosensory cortices during actual use of a prosthesis. Therefore, conclusions about the explanatory power of the embodiment hypothesis should be toned down.

We agree with this account, and do not wish to argue for a complete falsification of the embodiment theory, as we now extensively elaborate in the Discussion (see below). We have made additional efforts to moderated our interpretation of the results by further clarifying that any conclusive evidence presented against the embodiment hypothesis should be considered within the context of visual hand representation. We also offer numerous interpretations under which the embodiment hypothesis might still hold, within and outside of OTC. But we do not wish to through the baby out with the water - at the end of the day, our findings do empirically challenge (though not falsify) the embodiment theory and offer an alternative account for how prosthetic limbs are represented. Moreover, this alternative account correlates with prosthesis usage in daily life, a gold post that has never been successfully met before using neural embodiment measures. Up until now the embodiment hypothesis was the uncontested (and unproven) candidate of how the brain should ideally facilitate assistive technology, we therefore anticipate our finding to provoke an interesting discussion about alternative models for prosthesis representation in amputees.

Discussion (starting at page 15, 3^rd^ paragraph):

*“One potential solution originates from the fact that embodiment is a multifaced phenomenon [17], with distinct levels (i.e. phenomenological – does the prosthesis feel like my hand? cognitive – do I react to/with the prosthesis like I would to/with my own hand?; neural – is the prosthesis represented via the same mechanisms as a hand? [7]). […] .* ***It is possible, and even likely, that embodiment manifests differently in each of these distinct levels, which may also vary in their importance or even relevance for successful technological implementation [7]. To disentangle the complex concept of “embodiment”, future studies should aim to acquire measurements of the different levels of embodiment together with a detailed account of prosthesis skill and use.***

*A second important consideration is that embodiment is a multisensory process, involving multiple brain areas beyond visual cortex [41,56,57]. Here we focused on visuomotor processing, and our experimental approach does have some limitations that should be considered. For example,* ***our use of static images was designed to drive activity in the OTC, but not in other sensorimotor-related regions in the parietal and frontal regions [58], thereby limiting our conclusions to high-level visual cortex.*** *[…] .* ***Still, it is possible that other brain areas involved more directly in motor planning would produce other representational structures with respect to hand representation. Future research aimed at this question should take into consideration that at present, commercially available prosthesis motor control is fundamentally different from that of motor control of the hand, producing further potential challenges for sensorimotor embodiment****.*

*Thirdly, and most speculatively, it is possible that the prosthesis may still be represented in OTC as a body part, but one that is not a hand. […] . If body representation is not strictly tuned to the specific fine-grained features of a given body part (e.g. the digits of the hand), but is instead also represented at a higher level (e.g. effectors [60] or based on other functionality features [61]),* ***then the dissociation of prostheses from hand representation observed in our study should not be taken as evidence for lack of embodiment per se, but rather lack of embodiment as a hand****.* ***In this context the previously reported recruitment of hand-specific visual cortical regions, could reflect an underlying embodied representation of a prostheses as a (non-hand) body part****. Therefore, we propose that future studies of artificial limb embodiment should not be limited to identifying and/or quantifying hand representation (as the current common practice, e.g. using the rubber hand illusion).”*

A few more comments:
1.1 Currently, cosmetic and active prostheses are lumped together in most of the outcome measures. It could be the case that the embodiment hypothesis holds true only for one of them.
Supplementary Figure S3 is greatly appreciated to better understand the individual pairwise distances that make up the complex prosthesis similarity index which underlies the core argument of the manuscript - that the categorization hypothesis provides a better explanation of brain activity patterns than the embodiment hypothesis. Please provide statistical hypothesis tests for each of these displayed pairwise distances, and present this information in the main text. It is quite confusing to interpret the prosthesis similarity index without understanding the distances it is composed of. As the prosthesis similarity index is a key outcome measure, this is of paramount importance.

We thank the reviewer for this comment. While we had a clear hypothesis about the representational structure (interaction) that will lead to a categorisation effect, we did not have strong a priori predictions about the specific contribution of each pairwise distance. To address the reviewer’s query, we ran a data-driven approach (Principal Component Analysis; PCA), which we believe is highly suitable for exploring the key pairwise drivers of our findings. This is a more informative approach because it reveals the relative contributions of the representational structure across our five distance pairs, without limiting it to e.g. specific pairs. The results of this analysis are summarised in Table S4 of the revised manuscript.

While we would have liked to integrate this result in the main manuscript, due to strict image limitations of the Short report format (up to 4), this was not made possible. Instead we refer to it in the manuscript.

Results (page 9):

*“Exploratory data-driven analysis ran on the one-handers pair-wise distances comprising the Prosthesis-similarity index further revealed that the departure of the active prostheses in particular away from its native ‘tool’ category is linked with an increased association with cosmetic prostheses (See S4 Table).”*

Supplementary Materials (Table S4):

| **Pairwise distance** | **1^st^ component –  61.88% of variance** | **2^nd^ component –**  **18.43% of the variance** |
| --- | --- | --- |
| Hands <-> Active Prostheses | .873 | .025 |
| Hands <-> Cosmetic Prostheses | .823 | -.087 |
| Tools <-> Active Prostheses | .616 | **.725** |
| Tools <-> Cosmetic Prostheses | .907 | .025 |
| Cosmetic Prostheses <-> Active Prostheses | .674 | **-.622** |

***Supplementary Table S4. Principle component analysis (PCA) of pairwise distances.*** *To explore which of the pairwise distances contributed to the underlying observed effect of prosthesis categorisation we ran a data-driven analysis (PCA) on the five distances of the one-handed group. Values in the table are the weights given to each distance within a component. The 1^st^ component shows a ‘main effect’ of inter-individual differences across participants, in which some individuals have overall larger distances than others across all condition pairs. In our calculated indices we control for this effect by normalising individual’s selectivity indices by their Hands<-> Tools distance (See Methods). The 2nd component explains almost half of the remaining variance (after accounting for the inter-individual differences in component 1). In the 2^nd^ component, individuals showing greater distances between the active prostheses and the tool condition, also show greater similarity between the active prosthesis and the cosmetic prosthesis conditions. In other words, when the active prosthesis condition moves away from the tool category, it also tends to get closer to the cosmetic prostheses (as can be seen by the high weights and opposite signs of these two distances in the second component). This data-driven analysis provides further support for the hypothesised categorical shift of prosthesis representation.*

1.2 If the prosthesis similarity index should capture "degree to which the prostheses representation moved away from their natural categories (hands for cosmetic prostheses and tools for active prostheses) and towards one another", why does it include the pairwise distances hands <-> active prostheses and tools <-> cosmetic prostheses? It doesn't appear to be directly relevant under the above definition, and confuses the reader during their interpretation of the measure.

We take the reviewer’s point. Each of our indices included a prediction on the shift of each prosthesis with regards to both the hand and tool categories since we didn’t have a strong a-priori reason to predict the representational structure shift will be more pronounced in one direction or the other. But looking at the distances after-the-fact, we don’t see much differences in the mentioned distances: hands <-> active prostheses and tools <-> cosmetic prostheses. Following the reviewer’s comment, we recalculated the prosthesis similarity index without these two distances. This produced very similar results to the ones reported in the paper. A significant group effect when comparing the index between controls and prosthesis users results in: t(54)=-2.40, p=0.02; and a trend towards significance in the correlation with prosthesis usage: r(30)=0.33, p=0.07. While we agree with the reviewer’s feedback that these additional distances might unnecessarily complicate the results, we feel committed to transparently reporting our original analysis, in an effort to reduce flexible analyses (Makin & de Xivry, eLife, 2019).

Reviewer #2: Excellent revision. The manuscript has been much improved and it will now make a very valuable contribution to the literature. The results are fascinating. I just have a few minor comments.

2.1. Introduction, page 2. The term "visual embodiment" is new to me. Can we you define the term and/or give a reference the first time you use it on page 2.

We now provide a definition to the term in the introduction as follows:

*“Here we focus on visual embodiment* ***— the successful allocation of visual hand-related neural resources.****”*

2.2. Introduction, page 2. The phrase "visual embodiment has been suggested to the gateway for the sense of bodily ownership" is jargon. **Please provide a better explanation for why visual information is important for embodiment and body perception**. From a multisensory perceptive vision is important for localizing and identifying the limbs in space because the signal is spatially more precise compared with other senses (under good viewing conditions). I recommend you cite studies from the multisensory literature.

In the aforementioned manuscript, Tsakiris argues that bottom-up multisensory integration, emphasised by the reviewer, is not sufficient to induce bodily ownership. He argues that while sensorimotor information (‘body schema’) is continuously updated as the body moves, visual information of the object’s form and location is more constant, and may even relate to an internal model of what it means to be a body part. This sentence has now been rephrased to better covey this idea. Although if this sentence is still too inflammatory we are very happy to remove it altogether

We agree with the reviewer’s point (which is also reviewed in Makin et al., 2008, which we cite when discussing embodiment as a multisensory experience). But considering the present format (Short Report) we are not able to cover this rich literature more comprehensively. Instead, in the discussion we mention a couple of the references suggested by the reviewer.

Introduction (page 2, 2^nd^ paragraph):

*“Moreover, visual internal models of the body have been suggested as essential gateways for processing multisensory integration for successful bodily ownership [19], a desired feature of prosthesis usage [9,10].”*

Discussion (page 15, 2^nd^ paragraph):

*“A second important consideration is that embodiment is a multisensory process, involving multiple brain areas beyond visual cortex [41,56,57].”*

2.3. In their response letter, the author write that some authors have argued that the rubber hand is not embodied at a sensorimotor level during the rubber hand illusion, but rather at the visual level. I disagree with this view and argue that most researchers working on the rubber hand illusion probably describe this phenomenon as a multisensory body illusion rather than a visual illusion. The somatosensory (tactile-proprioceptive) aspects of the illusion are very prominent and the illusion works well without visual feedback of a limb (Ehrsson et al. 2005 J Neurosci; Guterstam et al. 2013 J Cog Neurosci).

We thank the reviewer for this feedback, and we fully take this point. Since our manuscript concerns visual embodiment and specifically that of prosthetic limbs, we focus on the common visual practice of the illusion, specifically involving visual manipulations. In the manuscript we only relate to the RHI in this context once, as follows:

Introduction (page 2, 2^nd^ paragraph):

*Previous efforts to test visual prosthesis embodiment have been centred on an illusion of body ownership over the prosthesis (most commonly, the rubber hand illusion, which relies on visual manipulations) […].”*

2.4. Page 3 and discussion. Most importantly, when the authors discuss OTC and EBA it is probably relevant to mention that this region respond to the rubber hand illusion, over and above tactile stimulation or the presentation of a visual hand image. The two best citations are probably Limanowski and Blankenburg (2014) Neuroimage (see also Limanowski and Blankenburg 2016 J Neurosci) that used an EBA localizer, and Gentile et al. (2013) J Neurosci that showed a correlation between LOC activity and the strength of the ownership illusion and who also found significant changes in functional connectivity between LOC and IPS (stronger PPI during hand ownership). This literature does not only suggest that OTC/EBA respond to tactile stimulation and has cross-modal properties, but actually, that this region play an important role in the key multisensory integration mechanisms related to body ownership/embodiment. I think this point is relevant for current study.

Absolutely, we thank the reviewer for these suggestions. These references have now been embedded in the introduction as follows:

Introduction (page 3, 2^nd^ paragraph):

*“OTC has been previously implicated in multisensory integration processing related to embodiment [39–41] and OTC’s connectome associates it with hand actions.”*

2.5. Page 14, first sentence, second paragraph. What is the difference between "neural visual embodiment" and "visual embodiment"? You have not registered "visual embodiment" behaviorally in this study, so we have no data about a possible relationship between visual embodiment and prothesis-use from a behavioral perspective. Please clarify this section.

We apologise if our phrasing was confusing. In the introduction we define what we mean by both neural and visual embodiment. For other reasons, the mentioned sentence has been removed from the revised manuscript. In the revised manuscript, when appropriate, we made sure to use the full term - neural visual embodiment.

2.6. Page 14, fourth paragraph, "other sensorimotor-related regions in the parietal and frontal regions (53)". I recommend that the authors to cite a couple of relevant original articles here (e.g. Gentile et al 2013), and  a more up-to date-review, for example Ehrsson (2020).

Ehrsson HH. Multisensory processes in body ownership. In: Sathian K, Ramachandran VS, eds. Multisensory Perception: From Laboratory to Clinic. Academic Press: Elsevier; 2020:179-200.
<https://eur01.safelinks.protection.outlook.com/?url=http%3A%2F%2Fwww.ehrssonlab.se%2Fpdfs%2FEhrsson%2520HH%25202020%2520Multisensory%2520processes%2520in%2520body%2520ownership.pdf&amp;data=02%7C01%7C%7Cd189c41bf00f42c4e5d108d7a9a61ba5%7C1faf88fea9984c5b93c9210a11d9a5c2%7C0%7C1%7C637164403209024009&amp;sdata=NZSGy%2BGhjDbU0VZoekD61%2B4R8xb1we20sEHnIoGGKbQ%3D&amp;reserved=0>

Thank you for these suggestions. We have now added the two mentioned references:

Discussion (page 15, 2^nd^ paragraph):

*“A second important consideration is that embodiment is a multisensory process, involving multiple brain areas beyond visual cortex [41,56,57].”*

**Reviewer #3**: The authors have done a meticulous job addressing my comments. I think a few minor issues may require further considerations.
The number below relates to my original comments.

3.1. I believe it would be better to formalise the 3rd possibility (that prosthesis anchor more into the representation of tools in prosthesis user) from the start- eg in the intro. As mentioned by the author this hypothesis is 'implicitly" announced and tested and I see that the results is confusing as others reviewers mention that prosthesis maps more like tools while the author in their response suggest that this is not statistically the case. Also, I think it makes sense to consider this possibility seriously since one of the main hypothesis for the development of tool selective region relate to tool-use and intrinsic connectivity between occipital-temporal and parietal regions (e.g. work of Stefania Bracci). I however let the author decide if they think this proposed strategy is best suited to present their work.

In the revisions, we highlight this important outcome at multiple paragraphs throughout the manuscript, starting with the results section. We feel that adding this consideration in the introduction might confuse the readers, who are already being introduced to two alternative hypotheses.

Results (page 7, 1^st^ paragraph):

*“****Notice that the Hand-similarity index is the inverse of a tool-similarity index. Therefore, significantly negative embodiment should be taken as evidence for association of the prosthesis with a tool****.* “

Results (page 9, 1^st^ paragraph):

*“This indicates that using a prosthesis alters one’s visual representation of different prostheses types into a distinct category, and does so in a way that is more complex than prostheses simply becoming more hand-* ***or tool-like****.”*

Discussion (page 15, 1^st^ paragraph):

*“Despite benefiting from hand-selective cortical resources [29], the prosthesis is not ‘embodied’ into people’s visual hand representation.* ***We also did not find any evidence for greater representation of prostheses as tools****.”*

Discussion (page 17, 1^st^ paragraph):

*“Instead of visual hand embodiment* ***(or tool-like representation****), we found that a significant amount of individual differences in prosthesis usage can be predicted by the extent of prosthesis categorisation within the visual body-selective area, providing a significantly better model than hand-embodiment.”*

Discussion (page 19, 2^nd^ paragraph):

*“Successful prosthesis usage, in terms of both wear-time and habit in daily life, was predicted not by visual embodiment (hand-similarity****) or tool-similarity****, but by a more distinct categorical representation of artificial limbs”*

3.2. I understand that the authors do not want to include these exploratory analyses in the manuscript. I would however be nice for the reader to get familiarised in the discussion with the idea that the visual expertise hypothesis, which is one potential reason behind the results of the current study, might be formally tested for instance by involving people highly involved in rehabilitative training of amputees.

We now added this consideration in our discussion as follows:

Discussion (page 17, 2^nd^ paragraph):

*“Since prosthesis usage relies on visuomotor expertise, it is difficult for us to disentangle the relative contribution of perceptual expertise to the observed prosthesis categorisation. Further research examining the representation of prosthesis in exclusively perceptual experts (e.g. prosthetists) will provide an interesting test case for our expertise hypothesis.”*

3.4. I am not sure the point was fully taken. What I was intending to question was the conceptual relationship of the current paper in the context of the Brain paper.
In the abstract of the Brain paper, one can read: "We show that the more one-handers use an artificial limb (prosthesis) in their everyday life, the stronger visual hand-selective areas in the lateral occipitotemporal cortex respond to prosthesis images." or, in the summary section "our findings show that neurocognitive resources devoted to representing our body can support representation of artificial body parts". This paper therefore suggest that images of prostheses anchor into visual hand selective region in prosthesis user. At first sight, this seems at odd with the conclusion of the current study showing that "prosthesis users represented their own prosthesis more dissimilarly to hands, compared to controls". I think that some text unifying the two studies may help more naive reader on the subject to create a coherent core of knowledge in this domain.

We wanted to revisit prosthesis representation in OTC, which we previously assessed using univariate activity, using a multivariate approach. Multivariate representational analysis provides a much more informative means to interrogate the activity pattern to directly examine its relevance to hand representation. In the introduction and the discussion, we detail how our result extend the previous finding and can deepen our understanding of it beyond a one-dimensional increase in activity level.

Introduction (page 3, 2^nd^ paragraph):

*We previously found that individuals who used their prosthesis more in daily lives also showed greater activity in OTC’s hand-selective visual areas when viewing images of prostheses, and greater functional coupling with sensorimotor hand areas [29]. This result demonstrates that prosthesis users are able to engage visuomotor hand-selective areas while passively representing a prosthesis. However, it is yet unknown whether prosthesis visual representation actually mimics that of a hand (i.e. neural embodiment). Alternatively, since the visual hand-selective regions partially overlap with hand-held tool representation [39], the observed activity gains may reflect the prosthesis being represented as a tool. As a third option, since object categorisation in OTC is thought to be based on their semantic and functional properties [40], expert prosthesis usage may result in the emergence of prostheses representation as a new ‘category’, diverging from its existing natural categories (e.g. ‘hands’, ‘tools’).*

Discussion (page 14, 1^st^ paragraph):

*“Previous research shows that prosthesis users are able to activate hand-selective areas in the OTC when viewing a prosthesis [29]. These areas, however, encompass a rich representational structure, spanning well beyond visual hand processing. It is therefore unknown whether by utilising these hand-selective areas, the users’ brain actually represents the prosthesis as a hand, or whether it follows a different representational principle.”*

In the revised manuscript we also further discuss an alternative account, where prosthetic limbs may be represented as body parts, though not like hands specifically. This interpretation might explain why prosthesis activity benefits from body-related OTC resources.

Discussion (page 15, 3^rd^ paragraph):

*“Thirdly, and most speculatively, it is possible that the prosthesis may still be represented in OTC as a body part, but one that is not a hand. After all, prosthesis users have strong semantic and sensorimotor knowledge of a hand (all users in the study had one intact hand; acquired amputees had prior experience of their missing hand, including lingering phantom sensations; see also [59] for related findings showing normal visual hand representation in individuals born without both hands). Their experience with operating a prosthesis is fundamentally different from that of a hand. If body representation is not strictly tuned to the specific fine-grained features of a given body part (e.g. the digits of the hand), but is instead also represented at a higher level (e.g. effectors [60] or based on other functionality features [61]), then the dissociation of prostheses from hand representation observed in our study should not be taken as evidence for lack of embodiment per se, but rather lack of embodiment as a hand.* ***In this context the previously reported recruitment of hand-specific visual cortical regions, could reflect an underlying embodied representation of a prostheses as a (non-hand) body part****.”*

**Reviewer #4**, Emily Graczyk: I have reviewed a previous version of the manuscript. I believe the current version is much improved and I thank the authors for their efforts to thoroughly address the points raised by reviewers. I have a few remaining comments that I would like the authors to consider.

Major points:
4.1. I thank the authors for clarifying that the focus of the article is on visual embodiment rather than sensorimotor embodiment. I think this clarification alleviates most of my previous major concerns. The authors' explanation of why visual embodiment was more appropriate to test in this study rather than sensorimotor embodiment was also helpful.
As another argument for the relevance of visual embodiment in one-handers, the authors note the over-reliance on visual guidance during normal prosthesis use. Could the authors comment on how this over-reliance on visual feedback when using prostheses could have influenced the results? Is it possible that the over-reliance on visual information could be a factor contributing to the differences in cortical representations of prostheses for prosthesis users compared to controls?

Indeed. More frequent visually-guided habitual prosthesis use (which strongly relies on visual feedback, especially when considering the limited somatosensory feedback afforded by artificial limbs) will increase processing for prostheses in the OTC visual areas and increase cross-talk between visual and sensorimotor hand-selective areas. This account feeds into a rich body of work relating to the role of visual expertise in shaping selectivity patterns in OTC. In the revised manuscript, we elaborate on this in the discussion as follows:

Discussion (page 17, 2^nd^ paragraph):

*“Since prosthesis usage relies on visuomotor expertise, it is difficult for us to disentangle the relative contribution of perceptual expertise to the observed prosthesis categorisation. Further research examining the representation of prosthesis in exclusively perceptual experts (e.g. prosthetists) will provide an interesting test case for our expertise hypothesis.”*

4.2. On page 8, the authors state "This indicates that using a prosthesis alters one's visual representation of different prostheses types into a distinct category, in between hands and tools…" It appears that the authors assume that the prosthesis category must be in between hands and tools along the hand-tool axis. In a multidimensional space, however, it's possible for the prosthesis category to be off of the hand-tool axis in a higher dimension. The calculations of the hand-similarity index and the prosthesis-similarity index do not appear to necessitate a single axis along which all three categories (hand, tool, prosthesis) must lie. I believe this implicit assumption of a single axis could influence the interpretation of the study by readers and should be removed.

As a way to address this point, it would be interesting to see an analysis breaking down the hand similarity index and the prosthesis similarity index into their component distance contributors. For example, how much of the differences between controls and prosthesis users in the prosthesis similarity index are due to the hand-cosmetic distance increasing vs the tool-active prosthesis distance increasing vs the cosmetic-active distance decreasing? Based on the way the prosthesis similarity index is calculated, any one of these distance changes could result in a higher prosthesis similarity index without all three distances necessarily changing. A discussion about the contributions of the inter-condition distances to the similarity indices and the extent to which the prosthesis category is on or off the hand-tool axis would be helpful.

We fully take this point. The categorisation index was formulated with this idea in mind, but our results in no way test for a single axis vs. multidimensional shift. We have removed all mentions of this interpretation when discussing the results.

To explore the relative contributions of inter-condition distances, we ran a data-driven analysis (Principal Component Analysis; PCA), which we believe is highly suitable for exploring the key pairwise drivers of our findings. This is an informative approach because it reveals the relative contributions of the representational structure across our five distance pairs, without limiting it to e.g. specific pairs. The results of this analysis are summarised in Table S4 of the revised manuscript.

While we would have liked to integrate this result in the main manuscript, due to strict image limitations of the Short report format (up to 4), this was not made possible. Instead we refer to it in the manuscript.

Results (page 9):

*“Exploratory data-driven analysis ran on the one-handers pair-wise distances comprising the Prosthesis-similarity index further revealed that the departure of the active prostheses in particular away from its native ‘tool’ category is linked with an increased association with cosmetic prostheses (See S4 Table).”*

Supplementary Materials (Table S4):

| **Pairwise distance** | **1^st^ component –  61.88% of variance** | **2^nd^ component –**  **18.43% of the variance** |
| --- | --- | --- |
| Hands <-> Active Prostheses | .873 | .025 |
| Hands <-> Cosmetic Prostheses | .823 | -.087 |
| Tools <-> Active Prostheses | .616 | **.725** |
| Tools <-> Cosmetic Prostheses | .907 | .025 |
| Cosmetic Prostheses <-> Active Prostheses | .674 | **-.622** |

***Supplementary Table S4. Principle component analysis (PCA) of pairwise distances.*** *To explore which of the pairwise distances contributed to the underlying observed effect of prosthesis categorisation we ran a data-driven analysis (PCA) on the five distances of the one-handed group. Values in the table are the weights given to each distance within a component. The 1^st^ component shows a ‘main effect’ of inter-individual differences across participants, in which some individuals have overall larger distances than others across all condition pairs. In our calculated indices we control for this effect by normalising individual’s selectivity indices by their Hands<-> Tools distance (See Methods). The 2nd component explains almost half of the remaining variance (after accounting for the inter-individual differences in component 1). In the 2^nd^ component, individuals showing greater distances between the active prostheses and the tool condition, also show greater similarity between the active prosthesis and the cosmetic prosthesis conditions. In other words, when the active prosthesis condition moves away from the tool category, it also tends to get closer to the cosmetic prostheses (as can be seen by the high weights and opposite signs of these two distances in the second component). This data-driven analysis provides further support for the hypothesised categorical shift of prosthesis representation.*

4.3.      The authors discuss the present findings in relation to two of their prior studies that appear to present contradictory or unexpected conclusions. The authors should provide a bit more discussion about how to rectify or interpret these different findings. First, the authors cite a prior study in the introduction showing that greater prosthesis use in daily life correlated to greater activity in OTC. However, the first paragraph of the results indicates that overall activity levels in the selected ROI were not different between prosthesis users and controls. This seems to contradict the previous results cited in the introduction and warrants further discussion. In addition, what are the implications of greater overall activation of OTC with increased prosthesis use on the inter-group comparisons in this study?

Second, the authors discuss a prior study showing that cognitive and phenomenological embodiment correlates to prosthesis usage. While this does not contradict the present findings that neural embodiment does not correlate to prosthesis usage, it does suggest that cognitive embodiment is not a consequence of neural embodiment in the visual cortex. Instead, it suggests that cognitive embodiment must arise from neural representations in some other brain area. A bit more discussion about these points would be helpful in interpreting the present work in the context of prior literature.

Regarding the reviewer’s first point:

The reported lack of significant univariate group differences reported here was meant as a methodological detail, since univariate effects could theoretically impact even noise-weighted multivariate distances (e.g. increased signal-to-noise will increase the pairwise distances). The two studies used different analysis approaches and different ROIs. We find that our multivariate categorical effect captures a more general organising principle of prosthesis representation in the OTC than univariate activity level, as it does not depend on the specific ROI definition in OTC. We appreciate this might be confusing, so we moved the line reporting no univariate differences to the methods.

Methods (page 24, Representational Similarity Analysis):

*“To assess the hand-prosthesis-tool representation structure within the ROI, pairwise distances between conditions were calculated using a multivariate approach, generally known as Representational Similarity Analysis [102]. Prior to performing the multivariate analysis, we first examined differences in univariate activity in the ROI, which could drive differences in the multivariate analysis. When comparing activity levels (averaged across all voxels) between controls and prosthesis-users within this region, no significant differences were found for each of the image conditions of hands, tools and prostheses (p>0.1 for all, see S2 Table), indicating that the two groups did not activate this region significantly differently. We then continued with the multivariate analysis.”*

Regarding the reviewer’s second point:

With respect to our cognitive study (which is now available on Biorxiv, Maimon Mor et al., 2020). Unfortunately, we are unable to directly compare how these two distinct findings interact, as they involved different participant groups. One possibility, as the reviewer suggest, is that these processes are orthogonal and mediated by different brain mechanisms. We mentioned this possibility in our discussion:

Discussion (page 14, 3^rd^ paragraph)

*“One potential solution originates from the fact that embodiment is a multifaced phenomenon [17], with distinct levels (i.e. phenomenological – does the prosthesis feel like my hand? cognitive – do I react to/with the prosthesis like I would to/with my own hand?; neural – is the prosthesis represented via the same mechanisms as a hand? [7]). In another recent study where we probed the phenomenological and cognitive levels of prosthesis embodiment, we found both to correlate with prosthesis usage [28]. Here we focused our research on the neural level, since it is entirely unknown whether objective representation of the prosthesis as a body-part associates with prosthesis acceptance and usage, let alone benefits it. It is possible, and even likely, that embodiment manifests differently in each of these distinct levels, which may also vary in their importance or even relevance for successful technological implementation [7].* ***To disentangle the complex concept of “embodiment”, future studies should aim to acquire measurements of the different levels of embodiment together with a detailed account of prosthesis skill and use****.”*

Discussion (page 14, end of 1^st^ paragraph)

*“Despite benefiting from hand-selective cortical resources [29], the prosthesis is not ‘embodied’ into people’s visual hand representation. We also did not find any evidence for greater representation of prostheses as tools.* ***However, since the experimental design and analysis we implemented were a priori focused on visual hand representation in OTC, it is possible that other brain areas might find a different representational ‘solution’ to support the daily use of an artificial limb****.”*

A second interpretation, which we have mentioned in our discussion (page 15, last paragraph) is that our notion of embodiment might be too simplistic, and that the prosthesis might be embodied as a body part that is not a hand. If true, the hand-similarity index is no longer the right measure for neural embodiment. It is possible that the newly formed prosthesis category is an embodied representation of prosthesis as a body part. Therefor it is possible that the categorical process we observe in OTC would contribute or even facilitate ‘cognitive’ embodiment (in this case spontaneous co-speech gestures with the prosthesis).

Discussion (page 15, last paragraph):

*“Thirdly, and most speculatively, it is possible that the prosthesis may still be represented in OTC as a body part, but one that is not a hand. After all, prosthesis users have strong semantic and sensorimotor knowledge of a hand […]. Their experience operating a prosthesis is fundamentally different from that of a hand. […]* ***In this context the previously reported recruitment of hand-specific visual cortical regions, could reflect an underlying embodied representation of a prostheses as a (non-hand) body part.”***

4.4.      I believe an analysis comparing the data from congenital and acquired one-handers is needed to support the authors' claim that the relationships do not depend on "visual mimicry of the human hand and cause of limb loss" (page 17). It appears that the data is presented separately for congenital and acquired one-handers in Figures 3 and 4, but there do not appear to be any statistical analyses presented that explicitly compare the two groups of one-handers. Were there any differences between congenital and acquired one-handers in hand similarity index or prosthesis similarity index? Were the relationships between prosthesis similarity index and prosthesis usage the same for congenital and acquired amputees?

The mentioned analysis of the influence of cause of limb-loss on our reported categorisation effect was already in the manuscript under the section: “Prosthesis-like (categorical), and not hand-like (embodied) representation of prostheses in prosthesis users”. To make sure this important analysis is not easily missed we have now moved it to the following section which is now called: “Prosthesis categorisation doesn’t depend on users’ developmental period of hand loss or prosthesis type” We are now also reporting a non-significant cause of limb-loss effect on the hand similarity index.

Results (page 10, Prosthesis categorisation doesn’t depend on users’ developmental period of hand loss or prosthesis type):

*“When considering hand-selective neural resources, individuals who were born without a hand might develop different representational structures than those with an acquired amputation [50]. Considering this, we tested whether the Hand-similarity index differed between the two prosthesis users sub-groups (congenital versus acquired), and found no significant differences (t(30)=-.615, p=0.54). Moreover, the reported Prosthesis-similarity effects prevailed even when accounting for any potential differences between the two sub-groups, as demonstrated by an analysis of covariance (ANCOVA) with sub-group (congenital vs acquired) as a fixed-effect and usage as a covariate. The ANCOVA revealed no significant sub-group effect (F(1,29)=2.02, p=0.17) – demonstrating that Prosthesis-similarity does not significantly differ due to cause/developmental period of hand-loss; and a nearly significant usage effect (F(1,29)=4.11, p=0.052) – indicating that the relationship between prosthesis categorisation and usage is relatively independent from potential sub-group effects.”*

4.5.      I think the manuscript was improved by the additional analyses focused on ROIs outside of EBA, and I thank the authors for their efforts on this. However, I think the interpretations stated in the section "Prosthesis representation beyond EBA" are stated too strongly. The only significant differences shown in IPS between prosthesis users and controls was for the "own prosthesis" condition. The hand and prosthesis similarity indices did not differ between controls and prosthesis users in the IPS. While I do not think this point weakens the overall study, I think it should be made clear that prosthesis categorization may be weaker in IPS.  I think this is further support for future investigations into neural representations of embodiment in other brain areas, as the authors suggest in the discussion.

We take this point, and have reworded this section.

Results (page 13, Prosthesis representation beyond EBA section):

*“With respect to the representational structure, we did not find significant differences between prosthesis users and controls when comparing both the Hand- and Prosthesis-similarity indices, even when wavering corrections for multiple comparisons which are customary for exploratory analysis (Hand-similarity: t(54)=0.71, p=0.48; Prosthesis-similarity: t(54)=-0.45, p=0.66).* ***This could be due to insufficient power to explore this representational structure, or possibly due to a different organising principle in this region****.”*

We also reworded the summary sentence of this paragraph to more accurately reflect the positive (significant) findings:

Results (page 12, Prosthesis representation beyond EBA section):

*“To demonstrate that our results are not specific to our ROI definition in OTC of EBA, we’ve repeated the same analysis, and found similar results, in ‘hand’ and ‘tool’ ROIs within OTC, generated from the meta-analysis tool Neurosynth [51] (see S1 Text and S4 Fig).*

*We next explored prosthesis representation beyond OTC. […]. Nevertheless, as demonstrated in S5A Fig, the visual stimuli also significantly activated the intraparietal sulcus (IPS), providing us with an opportunity to explore visual prosthesis representation in a dissociated brain area (though notably, this was observed less consistently within individual subjects). […]. However, we did find that users showed significantly greater dissimilarity (greater distances), relative to controls, when comparing the representation of their own prosthesis to that of both hands and tools (hand: t(25)=10.11, p<0.001; tool: t(25)=4.62, p<0.001, corrected for 2 comparisons, see S5C Fig).* ***This analysis indicates that in parietal cortex, similar to the OTC, users’ own prostheses are less similar to hands and tools, providing further support against the embodiment hypothesis****.”*

Unfortunately, we are not able to draw strong conclusions for the lack of prosthesis categorisation in IPS. This is because the design was not optimised for studying the parietal cortex, and because one should not over-interpret a null result (Makin & de Xivry, eLife, 2019). We have been running further studies which are better suited to address the role of parietal cortex in visual prosthesis representation, which are currently still ongoing.

Minor points:
4.6.      I thank the authors for clarifying why the task was designed to be passive. However, I have a few remaining questions about the images viewed by participants while in the scanner. The authors state that they used generic hand images for the task. Please clarify if the hands were matched in size, gender, and/or skin color to the participants' own hands. I think this detail needs to be explicitly stated in the methods or supplement, so that readers can understand the point the authors make in the discussion regarding the limitations of "generic hand images that are not the participants' own hands".

We have now specifically stated this in the methods (see below). We would like to turn the reviewer’s attention to the fact that all stimuli have been uploaded to Open Science Framework, for anyone who is curious about its specific characteristics. We think that seeing the images may be more informative than any textual description.

Methods (page 20, Stimuli section):

*“Participants viewed still object images of the following categories: (i) hands (upper limbs with and without the arm, in multiple orientations and configurations, and of different sizes, genders, and skin-colors; hereafter hands); …”*

4.7.      I thank the authors for the additional information about the combined prosthesis usage measure and the additional analyses demonstrating the relationships between the similarity indices and the PAL and wear time individually. In the previous revision, I asked the authors to provide evidence that the combined prosthesis usage measure had been validated. By validation of the metric, I mean is there data demonstrating internal consistency, minimal detectable change, and test-retest reliability of this metric? If this data is available, please indicate where it can be found. If not, please indicate this point in the methods.

We apologise for the misunderstanding. In Maimon-Mor et al, 2020 BioRxiv we include a test-retest reliability analysis for the two measures (PAL, wear-time frequency), measured for 21participants 1-2 years apart. Since the combined score is a sum of z-score transformation based on the specific dataset, we calculate the reliability of each measure separately. In short:

The PAL score was found to have good reliability with an ICC value of .81 (two- way random-model, absolute agreement type) and 95% confidence interval of single measures = .58 –.919 [F_(20,20)_ = 10.60 , p < .001]. For the wear time frequency, since it is an ordinal 6-item non-symmetrical scale we used Kendall’s tau-b correlation coefficient $\tau_{(19)}=0.605$, p=0.003. These analyses confirm that both measures have good consistency, providing validation for their use.

4.8.      In the abstract, the sentence beginning with "Moreover, prosthesis users represented their own prosthesis more dissimilarly to hands, compared to controls, challenging…" should be reorganized for clarity. It is difficult to distinguish what exactly was compared.
The mention sentence has now been rephrased:

Abstract:

*“Moreover, when comparing prosthesis users’ representation of their own prosthesis to controls’ representation of a similar looking prosthesis, prosthesis users represented their own prosthesis more dissimilarly to hands, challenging current views of visual prosthesis embodiment.”*

4.9      Page 12 - the acronym RSA is introduced and not defined.

This has now changed to read: “representational structure” to match the rest of the text.

4.10      Where was the prosthesis observation log (POL) score for control participants (shown in Table S1) used in this study? I do not think Table S1 should be removed, but it is not clear whether POL was used in any prosthesis usage analyses.
We agree that this information is not relevant for the results, and we have now removed these columns from the table.

4.11      In the results section titled "Prosthesis representation doesn't depend on the users' prosthesis type", other key factors of prosthesis use were not considered when analyzing prosthesis categorization: level of injury (transradial vs transhumeral), duration of time since limb loss, congenital vs acquired, and duration of time using primary prosthesis. Given the potential role of visual learning on the results, the duration of time since starting to use their primary prosthesis may be especially important to investigate in the future.

When working with a clinical population there are always numerous demographic/clinical factors to consider. To limit the number of independent and potentially underpowered comparisons we try to a-priori specify a small number of factors for further analysis (Makin & de Xivry, eLife, 2019). But we absolutely agree that our specific interests might not represent everyone in the community. As a solution to balance our study needs with that of the community, we will upload our data to the Open Science Framework to allow anyone interested to easily perform these comparisons themselves.

Methods (page 26, Statistical analysis):

*To minimise flexible analysis which could lead to p-hacking [108], only a limited set of factors, pre-specified in the initial stages of the analysis or based on the reviewer’s comments, were tested in this manuscript. Further exploratory analysis on other recorded clinical factors can be conducted using the full demographic details: https://osf.io/kd2yh/*

For the reviewer’s personal benefit, below we report the suggested comparisons for her interest.

Congenital vs acquired one-handers (see comment 4.4 above)

Level of injury (22 transradiasl vs 10 transhumeral):

Comparing the prosthesis similarity indices of the two sub-groups directly revealed a possible trend toward significance: t(30)=1.73, p=0.09, however this is confounded by the fact that these two groups might differ in prosthesis usage. We ran an ANCOVA with prosthesis usage as a covariate and level of amputation as a fixed effect, which produced the following results: no effect of level of amputation: F(1,29)=1.2, p=0.28 and as we already observed in previous analyses a significant prosthesis usage effect : F(1,29)=4.74, p=0.04.

Duration of time since limb loss (note that for congenital one-handers this is their age):

We found no significant correlation between the prosthesis similarity index and time since limb-loss
r(30)=0.26, p=0.15. Using a general linear model with both time since amputation and prosthesis usage as regressors we found no significant effect for time since amputation: F(1,29)=0.34, p=0.565 and again, a significant prosthesis usage effect: F(1,29)=4.59, p=0.04.

4.12.      Page 10 - "A group comparison between users of the two prosthesis types revealed no significant effect." It is not clear what metric is being compared between users of cosmetic and active prostheses in this section.

This has been revised as follows.

*“Comparing the Prosthesis-similarity index of users of the two prosthesis types revealed no significant effect (t(20)=.055, p=.96).”*
