# Supplementary material for: Is an artificial limb embodied as a hand? Brain decoding in prosthetic limb users
Source: PLoS Biol. 2020 Jun 8;18(6):e3000729. doi: 10.1371/journal.pbio.3000729 (PMC7302856; doi:10.1371/journal.pbio.3000729)
Supplement: Attachment — Submitted filename: ResponseToReviewers.docx [file pbio.3000729.s013.docx]

Dear editorial team,

Following your instructions, we have made the following changes:

- A ReadMe file was added to <https://eur01.safelinks.protection.outlook.com/?url=https%3A%2F%2Fosf.io%2F4mw2t%2F&amp;data=02%7C01%7C%7Cb05b820dbcbe41bf1a3c08d7f2a4c595%7C1faf88fea9984c5b93c9210a11d9a5c2%7C0%7C0%7C637244661813249829&amp;sdata=%2F6DWiqfI8jMru4jZPLWnH9Mo%2FgZfoUMTOj7c%2BqRgl8s%3D&amp;reserved=0> that explains how the data can be used to reproduce the figures.
- We have provided a detailed Financial Disclosure statement in the submission form. Including grant numbers and URLs as appropriate, as well as the initials of the authors who received the funding. We have also added the statement 'The funders had no role in study design, data collection and analysis, decision to publish, or preparation of the manuscript' to the end of your Financial Disclosure statement on the submission form.

Kind regards,

Roni Maimon-Mor on behalf of all co-authors
